# Extensive identification of genes involved in congenital and structural heart disorders and cardiomyopathy

**DOI:** 10.1038/s44161-022-00018-8

**Published:** 2022-02-17

**Authors:** Nadine Spielmann, Gregor Miller, Tudor I. Oprea, Chih-Wei Hsu, Gisela Fobo, Goar Frishman, Corinna Montrone, Hamed Haseli Mashhadi, Jeremy Mason, Violeta Munoz Fuentes, Stefanie Leuchtenberger, Andreas Ruepp, Matias Wagner, Dominik S. Westphal, Cordula Wolf, Agnes Görlach, Adrián Sanz-Moreno, Yi-Li Cho, Raffaele Teperino, Stefan Brandmaier, Sapna Sharma, Isabella Rikarda Galter, Manuela A. Östereicher, Lilly Zapf, Philipp Mayer-Kuckuk, Jan Rozman, Lydia Teboul, Rosie K. A. Bunton-Stasyshyn, Heather Cater, Michelle Stewart, Skevoulla Christou, Henrik Westerberg, Amelia M. Willett, Janine M. Wotton, Willson B. Roper, Audrey E. Christiansen, Christopher S. Ward, Jason D. Heaney, Corey L. Reynolds, Jan Prochazka, Lynette Bower, David Clary, Mohammed Selloum, Ghina Bou About, Olivia Wendling, Hugues Jacobs, Sophie Leblanc, Hamid Meziane, Tania Sorg, Enrique Audain, Arthur Gilly, Nigel W. Rayner, IMPC consortium, Juan A. Aguilar-Pimentel, Lore Becker, Lillian Garrett, Sabine M. Hölter, Oana V. Amarie, Julia Calzada-Wack, Tanja Klein-Rodewald, Patricia da Silva-Buttkus, Christoph Lengger, Claudia Stoeger, Raffaele Gerlini, Birgit Rathkolb, Daniela Mayr, John Seavitt, Angelina Gaspero, Jennie R. Green, Arturo Garza, Ritu Bohat, Leeyean Wong, Melissa L. McElwee, Sowmya Kalaga, Tara L. Rasmussen, Isabel Lorenzo, Denise G. Lanza, Rodney C. Samaco, Surabi Veeraragaven, Juan J. Gallegos, Petr Kašpárek, Silvia Petrezsélyová, Ruairidh King, Sara Johnson, James Cleak, Zsombor Szkoe-Kovacs, Gemma Codner, Matthew Mackenzie, Adam Caulder, Janet Kenyon, Wendy Gardiner, Hayley Phelps, Rhys Hancock, Claire Norris, Michayla A. Moore, Audrie M. Seluke, Rachel Urban, Coleen Kane, Leslie O. Goodwin, Kevin A. Peterson, Matthew Mckay, Jenn J. Cook, Jacob P. Lowy, Michael McFarland, Joshua A. Wood, Brandon J. Willis, Heather Tolentino, Todd Tolentino, Michael Schuchbauer, Jason Salazar, Jennifer Johnson, Rebecca Munson, Abdel Ayadi, Guillaume Pavlovic, Marie-Christine Birling, Sylvie Jacquot, Dalila Ali-Hadji, Philippe Charles, Philippe Andre, Marie-France Champy, Fabrice Riet, Igor Vukobradovic, Zorana Berberovic, Dawei Qu, Ruolin Guo, Abigail D’Souza, Ziyue Huang, Susan Camilleri, Milan Ganguly, Hibret Adissu, Mohammed Eskandarian, Xueyuan Shang, Kyle Duffin, Catherine Xu, Kyle Roberton, Valerie Laurin, Qing Lan, Gillian Sleep, Amie Creighton, Lauri Lintott, Marina Gertsenstein, Monica Pereira, Sandra Tondat, Amit Patel, Maribelle Cruz, Alex Bezginov, David Miller, Wang Hy, Atsushi Yoshiki, Nobuhiko Tanaka, Masaru Tamura, Zhiwei Liu, Olga Ermakova, Anna Ferrara, Paolo Fruscoloni, Claudia Seisenberger, Antje Bürger, Florian Giesert, Genomics England Research Consortium, J. C. Ambrose, P. Arumu gam, R. Bevers, M. Bleda, F. Boardman-Pretty, C. R. Boustred, H. Brittain, M. J. Caulfield, G. C. Chan, T. Fowler, A. Giess, A. Hamblin, S. Henderson, T. J. P. Hubbard, R. Jackson, L. J. Jones, D. Kasperaviciute, M. Kayikci, A. Kousathanas, L. Lahnstein, S. E. A. Leigh, I. U. S. Leong, F. J. Lopez, F. Maleady-Crowe, M. McEntagart, F. Minneci, L. Moutsianas, M. Mueller, N. Murugaesu, A. C. Need, P. O‘Donovan, C. A. Odhams, C. Patch, D. Perez-Gil, M. B. Pereira, J. Pullinger, T. Rahim, A. Rendon, T. Rogers, K. Savage, K. Sawant, R. H. Scott, A. Siddiq, A. Sieghart, S. C. Smith, A. Sosinsky, A. Stuckey, M. Tanguy, A. L. Taylor-Tavares, E. R. A. Thomas, S. R. Thompson, A. Tucci, M. J. Welland, E. Williams, K. Witkowska, S. M. Wood, Marc-Phillip Hitz, Eleftheria Zeggini, Eckhard Wolf, Radislav Sedlacek, Steven A. Murray, Karen L. Svenson, Robert E. Braun, Jaqueline K. White, Lois Kelsey, Xiang Gao, Toshihiko Shiroishi, Ying Xu, Je Kyung Seong, Fabio Mammano, Glauco P. Tocchini-Valentini, Arthur L. Beaudet, Terrence F. Meehan, Helen Parkinson, Damian Smedley, Ann-Marie Mallon, Sara E. Wells, Harald Grallert, Wolfgang Wurst, Susan Marschall, Helmut Fuchs, Steve D. M. Brown, Ann M. Flenniken, Lauryl M. J. Nutter, Colin McKerlie, Yann Herault, K. C. Kent Lloyd, Mary E. Dickinson, Valerie Gailus-Durner, Martin Hrabe de Angelis

**Affiliations:** 1 Institute of Experimental Genetics, German Mouse Clinic, Helmholtz Center Munich (GmbH), German Research Center for Environmental Health, Neuherberg, Germany; 2 Department of Internal Medicine, Division of Translational Informatics and Center of Biomedical Research Excellence in Autophagy, Inflammation, and Metabolism, UNM Health Sciences Center and UNM Comprehensive Cancer Center, Albuquerque, NM USA; 3 Department of Rheumatology and Inflammation Research, Institute of Medicine, Sahlgrenska Academy at University of Gothenburg, Gothenburg, Sweden; 4 Novo Nordisk Foundation Center for Protein Research, Faculty of Health and Medical Sciences, University of Copenhagen, Copenhagen, Denmark; 5 Department of Molecular Physiology and Biophysics, Baylor College of Medicine, Houston, TX USA; 6 European Molecular Biology Laboratory, European Bioinformatics Institute, Wellcome Trust Genome Campus, Hinxton, UK; 7 Institut für Humangenetik, Technische Universität Munich, Munich, Germany; 8 Klinik und Poliklinik Innere Medizin I, Klinikum Rechts der Isar, Technical University of Munich, Munich, Germany; 9 Department of Congenital Heart Defects and Pediatric Cardiology, German Heart Center Munich, Technical University Munich, Munich, Germany; 10 DZHK (German Centre for Cardiovascular Research), partner site Munich Heart Alliance, Munich, Germany; 11 Experimental and Molecular Pediatric Cardiology, German Heart Center Munich, Technical University Munich, Munich, Germany; 12 DZHK (German Centre for Cardiovascular Research), partner site Munich, Munich, Germany; 13 Research Unit of Molecular Epidemiology, Institute of Epidemiology II, Helmholtz Zentrum Munich, Munich, Germany; 14 German Center for Diabetes Research (DZD), Neuherberg, Germany; 15 Czech Centre for Phenogenomics, Institute of Molecular Genetics of the Czech Academy of Sciences, Prague, Czech Republic; 16 Mammalian Genetics Unit and Mary Lyon Centre, Medical Research Council Harwell Institute, Harwell, UK; 17 The Jackson Laboratory, Bar Harbor, ME USA; 18 Mouse Biology Program, University of California, Davis, Davis, CA USA; 19 Université de Strasbourg, CNRS, INSERM, IGBMC, Institut Clinique de la Souris, PHENOMIN-ICS, Illkirch, France; 20 Department of Congenital Heart Disease and Pediatric Cardiology, University Hospital of Schleswig-Holstein, Kiel, Germany; 21 German Center for Cardiovascular Research (DZHK), Kiel, Germany; 22 Institute of Translational Genomics, Helmholtz Zentrum München, German Research Center for Environmental Health, Neuherberg, Germany; 23 Wellcome Centre for Human Genetics, Nuffield Department of Medicine, University of Oxford, Oxford, UK; 24 Oxford Centre for Diabetes, Endocrinology and Metabolism, Radcliffe Department of Medicine, University of Oxford, Oxford, UK; 25 Wellcome Sanger Institute, Wellcome Genome Campus, Hinxton, UK; 26 TUM School of Medicine, Technical University of Munich and Klinikum Rechts der Isar, Munich, Germany; 27 Institute of Molecular Animal Breeding and Biotechnology, Gene Center, Ludwig-Maximilians-University Munich, Munich, Germany; 28 The Centre for Phenogenomics, Toronto, Ontario Canada; 29 Lunenfeld-Tanenbaum Research Institute, Sinai Health System, Toronto, Ontario Canada; 30 SKL of Pharmaceutical Biotechnology and Model Animal Research Center, Collaborative Innovation Center for Genetics and Development, Nanjing Biomedical Research Institute, Nanjing University, Nanjing, China; 31 RIKEN BioResource Center, Tsukuba, Japan; 32 Cambridge-Suda Genomic Research Center, Soochow University, Suzhou, China; 33 Korea Mouse Phenotyping Consortium (KMPC) and BK21 Program for Veterinary Science, Research Institute for Veterinary Science, College of Veterinary Medicine, Seoul National University, Seoul, South Korea; 34 CNR Institute of Biochemistry and Cell Biology, Monterotondo, Rome, Italy; 35 William Harvey Research Institute, Charterhouse Square Barts and the London School of Medicine and Dentistry Queen Mary University of London, London, UK; 36 Institute of Developmental Genetics, Helmholtz Zentrum Munich, German Research Center for Environmental Health GmbH, Neuherberg, Germany; 37 Department of Developmental Genetics, TUM School of Life Sciences, Technische Universität Munich, Freising, Germany; 38 Deutsches Institut für Neurodegenerative Erkrankungen (DZNE) Site Munich, Munich, Germany; 39 Munich Cluster for Systems Neurology (SyNergy), Adolf-Butenandt-Institut, Ludwig-Maximilians-Universität Munich, Munich, Germany; 40 The Hospital for Sick Children, Toronto, Ontario Canada; 41 Université de Strasbourg, CNRS, INSERM, Institut de Génétique Biologie Moléculaire et Cellulaire, IGBMC, Illkirch, France; 42 Department of Surgery, School of Medicine, University of California, Davis, Davis, CA USA; 43 Department of Experimental Genetics, TUM School of Life Science, Technische Universität Munich, Freising, Germany; 44 Genomics England, London, UK; 45 William Harvey Research Institute, Queen Mary University of London, London, UK

**Keywords:** Genetic predisposition to disease, Cardiovascular genetics

## Abstract

Clinical presentation of congenital heart disease is heterogeneous, making identification of the disease-causing genes and their genetic pathways and mechanisms of action challenging. By using in vivo electrocardiography, transthoracic echocardiography and microcomputed tomography imaging to screen 3,894 single-gene-null mouse lines for structural and functional cardiac abnormalities, here we identify 705 lines with cardiac arrhythmia, myocardial hypertrophy and/or ventricular dilation. Among these 705 genes, 486 have not been previously associated with cardiac dysfunction in humans, and some of them represent variants of unknown relevance (VUR). Mice with mutations in *Casz1*, *Dnajc18*, *Pde4dip*, *Rnf38* or *Tmem161b* genes show developmental cardiac structural abnormalities, with their human orthologs being categorized as VUR. Using UK Biobank data, we validate the importance of the *DNAJC18* gene for cardiac homeostasis by showing that its loss of function is associated with altered left ventricular systolic function. Our results identify hundreds of previously unappreciated genes with potential function in congenital heart disease and suggest causal function of five VUR in congenital heart disease.

## Abstract (web summary)

In a multicenter research program coordinated by the International Mouse Phenotyping Consortium, Spielmann et al. analyze the cardiac function and structure in ~4,000 monogenic mutant mice and identify 705 mouse genes involved in cardiac function, 75% of which have not been previously linked to cardiac heritable disease in humans. Using the UK Biobank human data, the authors validate the link between cardiovascular disease and some of the newly identified genes to illustrate the resource value and potential of their mutant mouse collection.

## Main

Human cardiovascular disease (CVD) is multifactorial with genetic and exogenous contributors. Almost all areas of clinical adult and pediatric cardiology include many rare CVDs that are associated with complex underlying genetic etiologies. These genetic disorders include subsets of inherited arrhythmias, cardiomyopathies, vascular diseases and/or structural heart defects that present as heterogeneous phenotypes with variable penetrance and expression^1^.

Arrhythmias are caused by anomalies in the electrical conduction system of the heart that regulates and coordinates myocardial contraction. The severity of arrhythmias varies from clinically benign to life threatening, causing sudden cardiac death^2^. Cardiac myocytes are excitable cells that generate an action potential in response to a stimulus that elicits a contractile response. Phases of the cardiac action potential, such as the P wave, the PR interval, the QRS complex or the QT interval, reflect atrial and ventricular depolarization or repolarization and can be measured by surface electrocardiography (ECG). Thus, any changes in ECG intervals can indicate abnormalities in cardiac conduction or electrical instability of the heart muscle leading to cardiac arrhythmia.

Primary cardiomyopathies are diseases of the heart muscle often associated with dysfunction of the sarcomere’s myofibrillar proteins, actin and myosin. Clinical manifestations include systolic or diastolic dysfunction. Abnormalities on echocardiography, such as enlarged ventricular chambers (that is, dilatation), myocardial wall thickening (that is, hypertrophy) or left ventricular (LV) systolic dysfunction indicate pathologic myocardial remodeling and/or cardiomyopathies.

Structural heart defects are the most prevalent congenital condition in human infants and result from perturbations to normal cardiac development causing structural abnormalities of the heart itself, the adjacent arteries and veins or a combination of heart and cardiac vasculature^3^. For example, ventricular septal defect (VSD) is a developmental malformation of the interventricular septum, allowing blood flow between the lumens of the two ventricles. Isolated VSD^4^ occurs in approximately two to six of every 1,000 live births and accounts for more than 20% of all human congenital heart disease (CHD), making it the second most common congenital heart defect after bicuspid aortic valve defects^5^.

Complete understanding of the pathogenesis of human congenital heart defects depends on knowing the genes (or potential candidates) that can cause or contribute to its manifestation and severity, knowledge of the relative penetrance and expressivity of these genes and informative mammalian models to identify a particular variant’s mechanism of action^6^. While many genes have been associated with CHD, the detection rate for rare congenital structural heart defect variants is low, at about 50% in syndromic CHD and about 30% in nonsyndromic CHD^7^. A large proportion of CHD, particularly in severely affected individuals, occurs in families in which there is no history of CHD.

In this study, we used 3,894 single-gene-knockout mouse lines (null deletion alleles) produced by the International Mouse Phenotyping Consortium (IMPC) for unbiased discovery of genes corresponding to monogenic heart disease phenotypes. We identified structural and functional cardiac abnormalities in adult mice by clinical in vivo ECG and transthoracic echocardiography (TTE) and by ex situ iodine contrast high-spatial-resolution microcomputed tomography (micro-CT) imaging of embryo hearts to reproduce the effects of known genes and identify hitherto unknown genes that as null alleles corresponded to congenital monogenic cardiac rhythm disorders, cardiomyopathies and structural heart defects. However, we have not considered vascular diseases. Our study identified 705 genes associated with cardiac rhythm disorder, myocardial hypertrophy and/or ventricular dilation phenotypes, 70% (486) of which have not, to our knowledge, been previously linked to cardiac function or cardiac disease. We further investigated a subset of the 486 genes not yet associated with CHD for causal inferences.

## Results

### Cardiac abnormalities associated with null alleles for 705 genes

**Extended Data Fig. 1:**
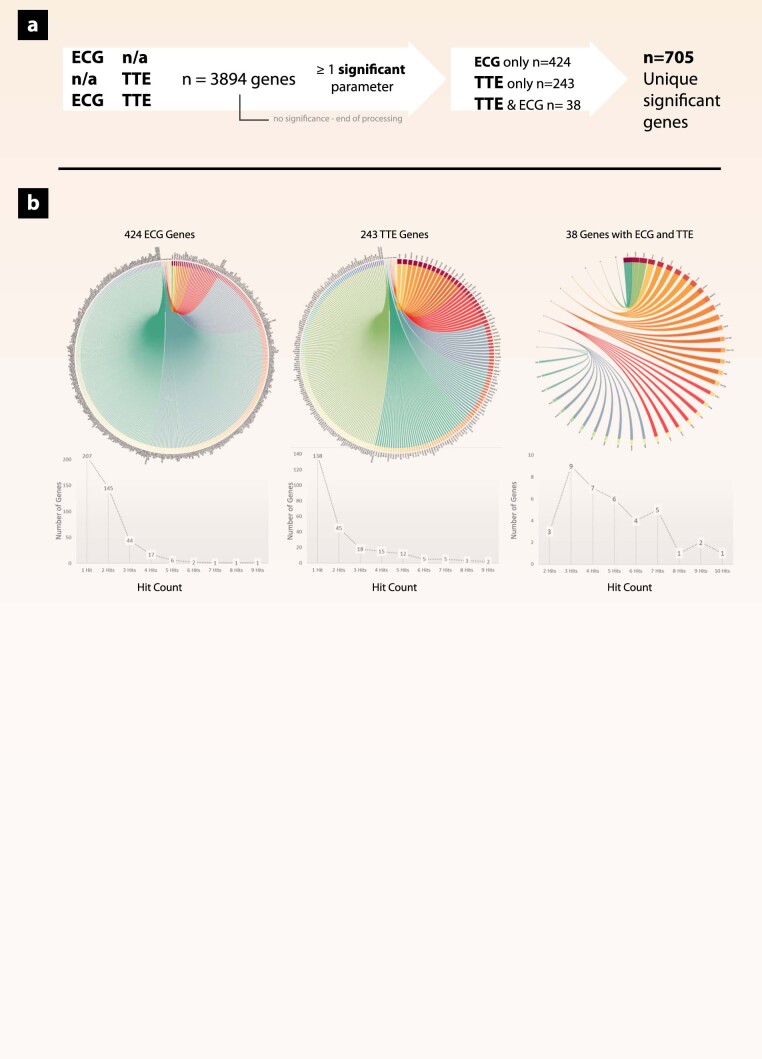
Summary of data gathering and genotype and phenotype information. a. Schematic overview of the IMPC data gathering. In total, 705 candidate genes with ≥ 1 significant parameter (q-value <0.05) in electrocardiography (ECG) or transthoracic echocardiography (TTE) derivates: ECG n = 424, TTE n = 243, TTE & ECG n = 38 target genes. b: Comprehensive representation of phenotype distribution across 705 genes. Genotype < >phenotype represented by chord graphs. Strength indicated by line thickness (thin lines - low phenotype count). Number of significant phenotypes (called ‘hits’) per gene (q-value <0.05). ECG - left, TTE – middle, both ECG and ECHO - right. Most genes with 1-2 hits, few with ≥ 3 hits. In gene knockouts with abnormal ECG, we observed an abnormal heart rate and inversely correlated RR interval duration in 29% (123/424) of mutant lines. A total of 24% of gene knockouts had QT alterations (102/424), 17% (72/424) in QRS, and 16% (68/424) in ST interval length. In the 243 knockouts with abnormal TTE data, we identified altered fractional shortening and ejection fraction associated with left ventricular dysfunction in 23% (56/243) of gene knockouts. Morphological differences in left ventricular interior diameter (LVID) or left ventricular posterior wall (LVPW) or anterior wall (LVAW) thickness observed in 20% (49/243) of the abnormal TTE knockouts. Half of the gene knockouts with abnormal cardiac phenotypes had a single abnormal phenotype; 49% (207/424) with ECG only and 57% (139/243) with TTE only compared to control animals.

We generated and analyzed phenotype data using TTE and ECG from 3,894 IMPC mouse lines homozygous for a single-gene null knockout mutation or heterozygous if homozygous mutation was lethal. All mutants and corresponding control animals were young adult mice (12 weeks of age) of the C57BL/6N background. This dataset included 3,836 of 3,894 lines with ECG data, 1,398 of 3,894 lines with ECG and TTE data and 58 lines with TTE data only. We identified 705 lines (genes) with at least one abnormal cardiac phenotype by applying the statistics of a soft windowing approach^8^ that compensates for temporal batch effect combined with *q*-value correction of the genotype contribution *P* values (R package qvalue^9^, version 2.14.1, R version 3.5.3 (ref. ^8^)) to account for multiple testing (Supplementary Data 1). The significance value for phenotyping deviance on these *q* values was set to 0.05. A total of 3,189 gene knockouts showed no significant differences in ECG and TTE parameters between mutants and controls. Separated by test, 424 gene knockouts had differences in ECG only between knockout mice and controls, 243 knockouts had differences in TTE only and 38 knockouts had significant differences for both tests (Extended Data Fig. 1a,b). A comprehensive overview of the null alleles and the detected phenotypes is provided in Supplementary Table 1. As examples, we focus here on five genes with the highest number of significant (that is, abnormal) parameters from a single test method, that is, five genes from ECG testing and five genes from TTE testing.

### Candidate genes for cardiac conduction system disease and rhythm disorders

**Fig. 1:**
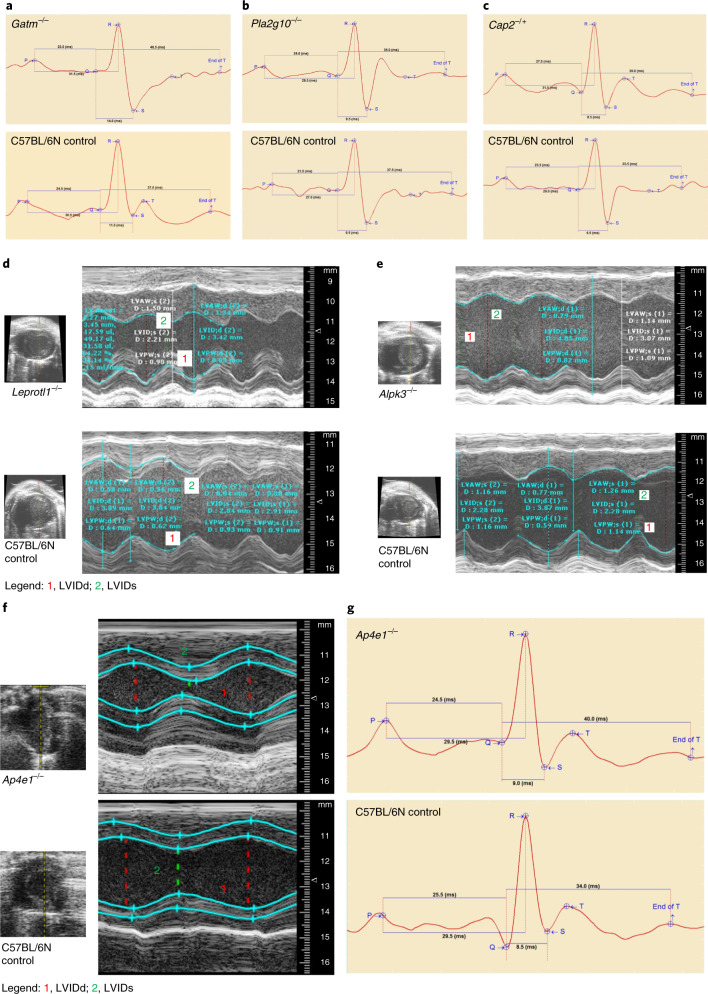
Candidate genes for cardiac conduction system disease and cardiomyopathy in mice. Representative electrocardiograms from conscious mutant and control mice with indication of ECG parameters and interval durations. **a**, *Gatm* loss (*Gatm*^−/−^) caused lower heart rate with prolonged QRS width and QTc and ST interval lengths. **b**, *Pla2g10* loss (*Pla2g10*^−/−^) lowered heart rate and prolonged PR, PQ and QTc intervals in female null mice. **c**, *Cap2* depletion (*Cap2*^−/+^) induced lower heart rate and lengthy QTc and ST durations in male null mice compared with C57BL/6N controls. Data are presented by Mouse Specifics software. Interval durations are given in milliseconds. M-mode recordings are through a short-axis view tangential to the papillary muscle from representative mutant and control mice. Images show the LVID throughout diastole and systole. **d**, *Leprotl1* depletion (*Leprotl1*^−/−^) reduced LV diameters (LVIDs and LVIDd) and increased myocardial wall thickness (LVAWs, LVAWd and LVPWs) with decreased systolic function compared with C57BL/6N controls. **e**, *Alpk3* depletion (*Alpk3*^−/−^) increased LV diameters (LVIDs and LVIDd) and decreased systolic function via reduced fractional shortening and ejection fraction, suggesting dilated left ventricle or even dilated cardiomyopathy. The *y* axis represents the distance (in mm) from the transducer (Vevo 2100); time (in ms) is on the *x* axis. **f**, *Ap4e1* loss (*Ap4e1*^−/−^) caused an impairment of LVIDd and LVIDs and consequently lowered stroke volume. The *y* axis represents the distance (in mm) from the transducer (Vevo 2100); time (in ms) is on the *x* axis. **g**, Representative electrocardiograms from conscious *Ap4e1*-mutant and C57BL/6N control mice with indication of ECG parameters and interval durations. *Ap4e1* loss lowered heart rate and concurrently increased RR interval duration. Data are presented by Mouse Specifics software.

Among 424 gene knockouts with ECG alterations, homozygous *Gatm*-, *Pla2g10*-, *Elmod1*- and *Masp1*-knockout mice and mice heterozygous for *Cap2* had the highest number of significant phenotype differences compared to controls. These five genes, given the presence of multiple abnormal ECG phenotype parameters, are most likely to be associated with abnormal function of the cardiac conduction system and cardiomyocyte arrhythmias. The gene *Gatm* (glycine amidinotransferase, l-arginine–glycine amidinotransferase) encodes a mitochondrial enzyme involved in creatine biosynthesis with a potential role in embryonic and central nervous system development^10^. Homozygous deletion of *Gatm* resulted in low heart rate with prolonged QRS width and rate-corrected QT (QTc) and ST interval length (Fig. 1a). *Pla2g10* encodes phospholipase A2 group 10, a calcium-dependent enzyme, which catalyzes the release of arachidonic acid from cell membrane phospholipids^11^. *Pla2g10* homozygous knockout mice had a lower heart rate with prolonged PR, PQ and QTc interval length, and females were predominantly affected (Fig. 1b). *Elmod1* encodes ELMO/CED-12 domain-containing 1, which acts as a GTPase-activating protein for the ARF family of small G proteins^12^. Homozygous deletion of *Elmod1* had no effect on heart rate but shortened the duration of PR, PQ and ST intervals. *Masp1* encodes mannan-binding lectin serine protease 1, a serine protease that functions as a component of the lectin pathway of complement activation with an essential role in innate and adaptive immune responses^13^. Homozygous deletion of *Masp1* resulted in lower heart rate with prolonged QRS and ST intervals. *Cap2* encodes adenylate cyclase-associated protein 2 (yeast) (CAP), a protein related to the *Saccharomyces cerevisiae* CAP protein involved in the cyclic AMP pathway that regulates filament dynamics and is implicated in a number of complex molecular and morphological developmental processes, including mRNA localization and the establishment of cell polarity^14^. Heterozygous deletion of *Cap2* caused a lower heart rate and prolonged QTc and ST durations, both indicative of ventricular conduction delay (Fig. 1c). None of these five genes with the highest number of significant differences in ECG parameters have previously been reported to be associated with cardiac conduction system disease and rhythm disorders.

### Candidate and known genes for monogenic cardiomyopathies

Of the 243 gene knockouts with significant TTE alterations, homozygous *Fzd8*-, *Leprotl1*-, *Abhd3*-, *Ints6l*- and *Alpk3*-knockout mouse lines had the highest number of significant abnormal phenotypes compared to controls. The gene *Fzd8* (encoding frizzled class receptor 8) is an intronless member of the frizzled gene family that encodes frizzled receptors that play a critical role when coupled to the canonical WNT–β-catenin pathway in LV remodeling and cardiac development^15^. Homozygous loss of *Fzd8* resulted in reductions of LV internal diameters (LVID) during systolic and diastolic contraction (LVIDs and LVIDd) with increased myocardial wall thickness (LV anterior wall (LVAW)s, LVAWd and LV posterior wall (LVPW)s) and decreased systolic function measured by fractional shortening and ejection fraction. In this study, all homozygous *Fzd8*-null mice showed distinct features of hypertrophic cardiomyopathy with normal ECG, strongly suggesting a role for *Fzd8* in cardiomyopathy. Identical hypertrophic cardiomyopathy phenotypes were identified in *Leprotl1* (encoding homozygous leptin receptor overlapping transcript-like 1) mutants compared to controls (Fig. 1d). The product of *Leprotl1* negatively regulates cell surface expression of the growth hormone (GH) receptor in the liver and is an important metabolic regulator stimulating lipolysis, preventing protein catabolism and reducing insulin-dependent glucose disposal during nutrient starvation. *Leprotl1* was also shown to be a molecular link between nutritional signals and GH activity that influences body growth and metabolism^16^. A hypertrophic cardiomyopathy phenotype is unrecognized for *Leprotl1*. By contrast, mice with homozygous loss of *Ints6l* (integrator complex subunit 6 like) or *Abhd3* (abhydrolase domain-containing 3) had an increase in LV diameters (LVIDs and LVIDd) with decreased systolic function, indicated by reduced fractional shortening and ejection fraction. These findings confirmed decreased thickness of both LVAW and LVPW, suggesting dilated LV cardiomyopathy in both mutant lines. A cardiomyopathy phenotype has not been reported previously for these two genes. The gene *Alpk3* (α-kinase 3) is involved in cardiomyocyte differentiation and is associated with several forms of cardiomyopathy^17^. Homozygous *Alpk3*-knockout mice had features of both hypertrophic and dilated forms of cardiomyopathy, reproducing the same phenotype previously reported for an *Alpk3* mouse model^18^. Our test data confirmed reproducible myocardial hypertrophy in *Alpk3*-null mice, characterized by increased end-diastolic LVAW and LVPW thickness. Several changes more typically associated with dilated cardiomyopathy included marked increases in LV end-diastolic and end-systolic volumes and increased LV diameters (LVIDs and LVIDd) as well as reduced ejection fraction and fractional shortening, suggestive of LV chamber dilation (Fig. 1e). Of the five representative examples with abnormal TTE reported here, four represent unidentified candidate genes for cardiomyopathy and one provides validation of a previously reported association.

Thirty-eight of the gene-knockout mouse lines that we tested had abnormal phenotypes as shown by both ECG and TTE. Heterozygous loss of smoothened, frizzled class receptor (*Smo*) resulted in prolonged PR and PQ in ECG, and, under isoflurane anesthesia, *Smo* mutants showed normal cardiac function and normal LV dimensions but a higher heart rate as shown by TTE. Other examples included scavenger receptor class B member 1 (*Scarb1*)^19,20^ and collagen type I α2 (*Col1a2*)^21–23^, known for their role in CVD, and adaptor-related protein complex AP-4 ε1 (*Ap4e1*) and LSM1 homolog, mRNA degradation associated (*Lsm1*), which our data identify as undetected candidates for cardiac defects. The product of *Ap4e1* is a component of the adaptor protein complex, the components of which are involved in both vesicle formation and cargo selection. AP-4-deficiency syndrome arises from defects in protein coats involved in intracellular protein sorting referred to as ‘coatopathies’, which are multi-systemic and often impact the central nervous system^24^. Homozygous loss of *Ap4e1* resulted in decreased heart rate, confirmed by increased RR intervals in electrocardiograms. *Ape4e1*-knockout mice also had impaired LV myocardial function with reduced stroke volume, indicating reduced volume of blood pumped out of the left ventricle during each systolic cardiac contraction. At the same time, LVIDs was reduced, but systolic function increased, indicated by greater fractional shortening and ejection fraction (Fig. 1f). A previously published *Ap4e1*-null mouse model had motor deficits and neurological defects, which are consistent with phenotypes observed in humans with AP-4-deficiency syndrome^24^.

### Candidate genes causing heart VSD

We used ex situ imaging of the embryo heart in homozygous lethal or homozygous subviable single-gene-knockout mice to identify structural heart defects. Of the mouse lines studied here, 65% were homozygous knockout mice (corresponding to loss of function (LOF) in human) and 35% were heterozygous knockout mice. We observed a variety of structural heart defects in mutants with lethal and subviable phenotypes but most frequently VSD. Homozygous single-gene deletion of *Sirt1*, *Stambp*, *Casz1*, *Wfdc2*, *Tmem161b*, *Nxn*, *Dnajc18*, *Gnao1* and *Slc25a1* resulted in substantial structural heart anomalies when compared to control littermate embryos. In young adult heterozygous mice, the same genes caused ECG abnormalities (*Sirt1*, *Stambp*, *Casz1* and *Wfdc2*) and abnormal TTE measurements (*Tmem161b*, *Nxn*, *Dnajc18*, *Gnao1* and *Slc25a1*). Complementary gross morphological examination of the embryos confirmed structural cardiac abnormalities in homozygous lethal knockout mice for five additional genes: *Zfp503*, *Ubr4*, *Furin*, *Smo* and *Shox2* (Table 1). In young adult heterozygous mice, the same genes caused ECG (*Zpf503*) or TTE alterations (*Ubr4* and *Furin*) or both ECG and TTE alterations (*Smo*) when compared to age- and sex-matched controls (Supplementary Data 1). Young adult heterozygous *Shox2*-mutant animals had not been tested by ECG or TTE at the time of publication; therefore, no adult data were available.

**Table 1:** In-depth characteristics of 14 genes included in the VSD network

Gene	Allele	Primary viability	Secondary viability	Adult TTE	Adult ECG	Embryo GM abnormalities	Embryo micro-CT VSD
*Sirt1*	tm1b	Subviable	Viable, E18.5	No hit	*rMSSD ↑*	Nervous system, eyes, immune system	
*Stambp*	tm1b	Subviable	Viable, E18.5	No hit	**QTc ↓**	All normal	
*Casz1*	tm1b	Lethal	Viable, E14.5	No hit	ST, QTc ↓	Bone, cartilage, muscle	
*Wfdc2*	tm1b	Subviable	Viable, E14.5	No hit	RR ↑	Respiratory system	
*Tmem161b*	tm1b	Lethal	Viable, E14.5	*Stroke volume, cardiac output ↑*	No hit	Nervous system, eye	
*Nxn*	tm1b	Lethal	Viable, E18.5	Ejection fraction, fractional shortening, HR (TTE) ↓	No hit	Craniofacial morphology, bone, cartilage, muscle	
*Dnajc18*	tm1b	Subviable	Viable, E18.5	LVIDd, **stroke volume, cardiac output** ↑	No hit	All normal	
*Gnoa1*	tm1b	Lethal	Viable, E18.5	*LVIDd, LVIDs, end-systolic diameter, end-diastolic diameter ↓*	No hit	Reproductive system	
*Scl25a1*	tm1b	Lethal	Viable, E18.5	LVAWd, LVAWs, cardiac output, HR (TTE) ↓	No hit	Abnormal embryo size, abnormal embryo development, embryonic growth retardation, pallor, abnormal facial morphology, exencephaly, abnormal head size, abnormal head shape, protruding tongue, abnormal limb morphology, abnormal tail morphology	
**Genes positive for abnormal heart morphology in the VSD network**							
**Gene**	**Allele**	**Primary viability**	**Secondary viability**	**Adult TTE**	**Adult ECG**	**Embryo GM abnormalities**	**Embryo micro-CT VSD**
*Smo*	tm1b	Lethal	Viable, E9.5–E12.5	HR (TTE) ↑	PQ ↑; HR ↓; CV, QTc dispersion, ST, rMSSD, RR, HRV, PR ↑	Abnormal embryo development, abnormal embryo turning	Abnormal pericardium morphology
*Ubr4*	tm1b	Lethal	Viable, E9.5	LVIDd ↓, ejection fraction ↑	No hit	None	Heart looping defects
*Furin*	tm1b	Lethal	Viable, E9.5	Ejection fraction, fractional shortening ↓; LVIDs ↑	No hit	Abnormal brain (forebrain, midbrain and hindbrain), eye morphology, neural tube	Aortic sac, ventricular chamber, atrioventricular canal, heart trabeculation, arterial chamber, dorsal aorta
*Shox2*	tm1b	Lethal	Lethal, E11–E13	No hit	No hit	Craniofacial defects, wavy spinal cords and edema, especially around the heart	Edema around the heart
*Zfp503*	tm1b	Subviable	Viable, E15.5	No hit	HR ↓, RR ↑	Sternum, left lung and right lung, cranial, accessory, caudal and middle lobe anomalies	Ductus arteriosus, pulmonary trunk, heart ventricle

Characterization of nine genes involved in VSD (top) and five genes associated with abnormal heart morphology (bottom) by target allele, primary and secondary viability, significant (*q* value set at 0.05) adult ECG and TTE phenotypes, gross morphology (GM) and microcomputed (micro-CT) visualization of fetal hearts (left, healthy control; right, mutant heart with structural VSD). Note that arrows indicate phenotype differences in knockout compared with C57BL/6N mice: increased, ↑; decreased, ↓. HR, heart rate; HRV, heart rate variability; CV, coefficient of variation; RR, interval between successive Rs;rMSSD, root mean square of successive differences; bold (male) and italic (female) text in adult TTE and ECG columns indicates sex effect.

**Fig. 2:**
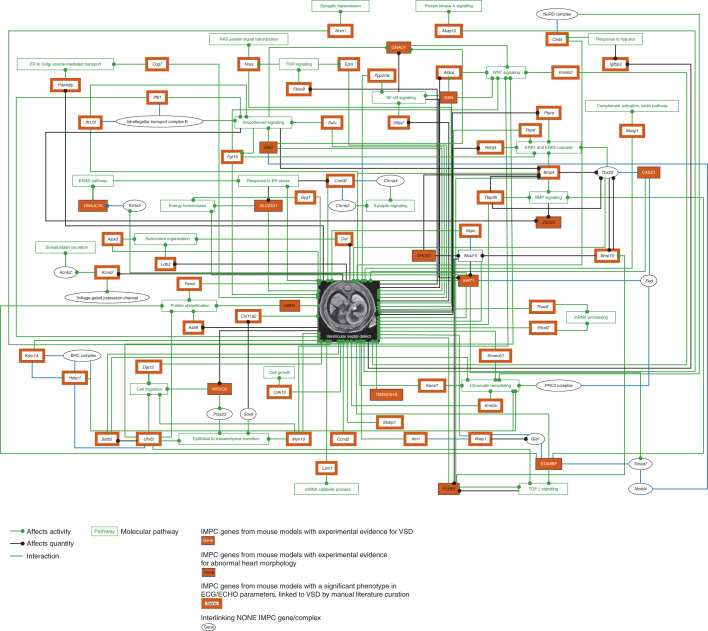
VSD network. Ex situ imaging of the embryo heart in homozygous lethal or homozygous subviable single-gene-knockout mice used to identify structural heart defects, such as VSD. Of the mouse lines studied here, 65% were homozygous knockouts (corresponding to LOF in human) and 35% were heterozygous knockouts. This dataset included 248 of 705 lines (35%). Lines were confirmed to be lethal or subviable; we assessed cardiac development by analyzing three-dimensional micro-CT data obtained from iodine contrast-enhanced micro-CT that provides high spatial resolution of up to 3–14 μm per voxel from embryonic day (E)9.5–E18.5 embryos (http://www.mousephenotype.org/data/embryo). No embryo imaging was performed on viable knockout lines. Embryo data were available for only a small subset of the total knockout genes used for this study. The VSD network included nine genes (*Sirt1*, *Stambp*, *Casz1*, *Wfdc2*, *Tmem161b*, *Nxn*, *Dnajc18*, *Gnao1* and *Slc25a*) that, when LOF was induced, caused early mortality due to structural heart changes but most notably VSDs in the null mutant mice, experimentally shown by computed tomography microscopy. Five more genes (*Zpf503* (human *ZNF503*), *Ubr4*, *Furin*, *Shox2* and *Smo*) were associated with cardiac abnormalities after gene depletion, confirmed by gross morphology data. An additional 45 genes were integrated using their association with cardiac malformations and development of a VSD. Only in vivo ECG and TTE data from young adult mice are available because these mutant lines tested positive for viability; therefore, no embryo screening was performed. The network analysis also identified 13 non-IMPC interacting genes. Two transcription factors, NKX2-5 (*Furin*, *Bmp10*, *Shox2*, *Sirt1* and *Sspn*) and TBX20 (*Bmp4*, *Bmp10*, *Tfap2b* and *Casz1*), known to be important for early cardiac development, were strongly represented. Furthermore, BMP10, a critical regulator of cardiac growth and chamber maturation, chromatin-modifying (*Hdac1* and *Smarcb1*) genes and genes regulated by processes essential for cardiogenesis, for example, smoothened and WNT signaling (*Gnao1*, *Emilin2*, *Aldoa*, *Nxn*, *Smo*, *Sufu* and *Ift81*), were strongly represented in the network. Compelling evidence of experimental human or rodent data is from relevant publications, primarily peer-reviewed ‘small-scale experiment’ literature used in the network analysis. ECHO, transthoracic echocardiography; ECG, electrocardiography; NONE, genes not analyzed so far in the IMPC; ER, endoplasmic reticulum.

Ex situ screening of embryonic hearts identified structural heart defects in mutants for genes previously associated with CVD (*Stambp*, *Nxn*, *Slc25a1*, *Smo* and *Shox2*) as well as hitherto unassociated candidate genes (*Sirt1*, *Casz1*, *Wfdc2*, *Tmem161b*, *Dnajc18*, *Zfp503* and *Ubr4)*. These 14 genes, all of which when deleted caused a structural VSD in the heart, were further investigated for common elements using CIDeR-based multifactorial interaction network analysis (http://mips.helmholtz-muenchen.de/cider^25^). This analysis identified associations for an additional 45 IMPC genes from our study and 13 interacting non-IMPC genes associated with structural heart defects (Fig. 2).

### Association of *Gnao1* with VSD

To exploit the utility of our VSD dataset, we enriched our deep literature curation and the automated text mining tool ‘Geneshot’ (ref. ^26^) with human VSD data. This analysis identified genes known to be linked to VSDs (*SIRT1* (ref. ^27^), *SLC25A1* (ref. ^28^) and *NXN*^28^) and genes with no direct human experimental evidence for VSDs (*TMEM161B*, *WFDC2, SHOX2*, *GNAO1*, *UBR4* (ref. ^29^) and *FURIN*^30^) apart from rare case studies that suggest association with structural heart defects for some of these genes. An example is one clinical report of a single patient with neurological disease and *a STAMBP* mutation with congenital malformations of the ventricular septum^31^. Further evidence is found in hypermethylation of the *WFDC2* promoter in fetal myocardia with VSDs^32^ and association of an *NKX2-5* variant, a gene suppressed by the regulator SHOX2, in a family with autosomal dominant inherited VSDs^33^ and cardiac arrhythmia^34^. GNAO1, the α subunit of the guanine nucleotide-binding protein, has no link to VSD but does have an association with infantile epileptic encephalopathy^35^, a neurological disorder not previously reported to be associated with congenital heart defects. Consequently, our data demonstrate that loss of *Gnao1* results in a structural heart defect (that is, VSD) in mice and it should henceforth be considered a VSD gene candidate.

### Unbiased large-scale cardiac phenotyping of knockout mouse lines identifies 486 unrecognized genes involved in cardiac function or development

**Fig. 3:**
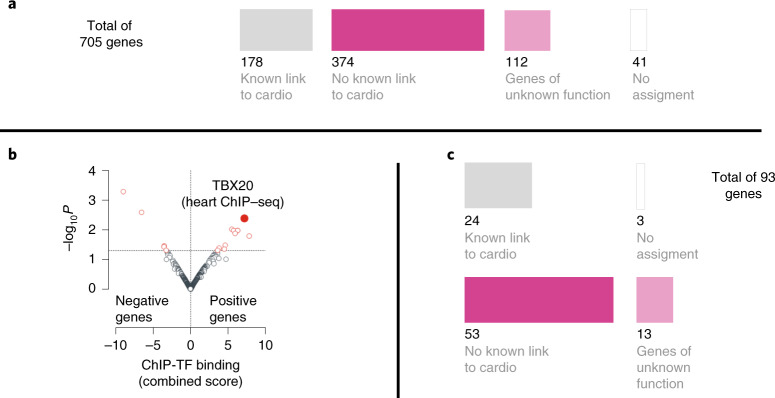
Alignment and enrichment of 705 IMPC knockout genes. Using Pharos (https://pharos.nih.gov/), a multimodal web interface, we queried alignment for human cardiac disease relevance. We excluded 41 genes that had no human ortholog and/or limited assignment information from this analysis. This analysis indicated that 155 of the 664 mouse–human orthologous genes were previously linked to heart disease. The remaining 509 orthologous genes had not been previously associated with a cardiac condition and likely represent ‘unappreciated candidate genes’ for CHD. This set of 509 genes was further queried for newness using the Online Mendelian Inheritance in Man (OMIM) catalog (https://omim.org/) and Orphanet, a rare disease portal (https://www.orpha.net/) dataset. This secondary analysis confirmed that 486 of 509 genes were unknown candidate genes and the remaining 23 genes have been associated predominantly with pleiotropic neurodevelopmental disorders that include sporadic congenital heart defects or malformations. **a**, Alignment of 705 IMPC knockout genes using Pharos, OMIM and Orphanet classified them into genes with a previously known link (178 of 705) or unknown link (486 of 705) to CVD or poorly annotated genes (112 of 486); cardio, cardiovascular diseases and/or heart. **b**, Genes previously identified with abnormal cardiac function in knockout mice (positive genes) showed strong enrichment (*P* = 0.004; odds ratio, 1.31; combined score, 7.2) for heart-specific targets of the transcription factor TBX20, a critical regulator of heart development^65–67,97^, associated with human CHD and adult cardiomyopathies^68,70,98,99^, while the 3,189 nonsignificant IMPC knockout genes (negative genes) did not. The ChEA 2016 dataset of publicly available ChIP–seq experiments was used for in silico analysis^42^. Here ‘strong’ enrichment was considered when the following parameters were met: combined score >5 and adjusted *P* value <0.05; TF, transcription factor. **c**, Molecular properties of TBX20 target genes showed that 66 of 93 (~70%) have no previous known link to CVD or are poorly annotated genes (13 of 93, ~14%). This observation indicates that, while the role of TBX20 in heart physiology is not completely understood, our dataset sheds light on a previously unknown group of genes with potential relevance for heart development and function.

To distinguish genes with a known association with human heart abnormalities from genes with no previously published association, we used comprehensive upstream literature-based Pharos (https://pharos.nih.gov/^1^) analysis of all 705 genes irrespective of the test modality and type of phenotype abnormality that we identified. This analysis indicated that 178 of the 664 mouse–human orthologous genes (41 nonorthologous genes) were previously linked to heart disease, some of which functionally converge on skeletal and cardiac muscle structural integrity (for example, *LDB3*)^36^, cardiac development (*BMP10*)^37^, calcium signaling (*CACNB2* and *DSG2*)^38,39^, sodium channels (*SCN2B*)^40^ and sarcomere contraction cycle (*TNNC1* and *MYH2*)^30,41^. Notably, 486 orthologous genes had not been previously associated with a cardiac condition and likely represent unappreciated candidate genes for CVD and more yet for CHD (Fig. 3a).

### Mouse genes associated with CHD phenotypes are often regulated by the transcription factor TBX20

**Extended Data Fig. 2:**
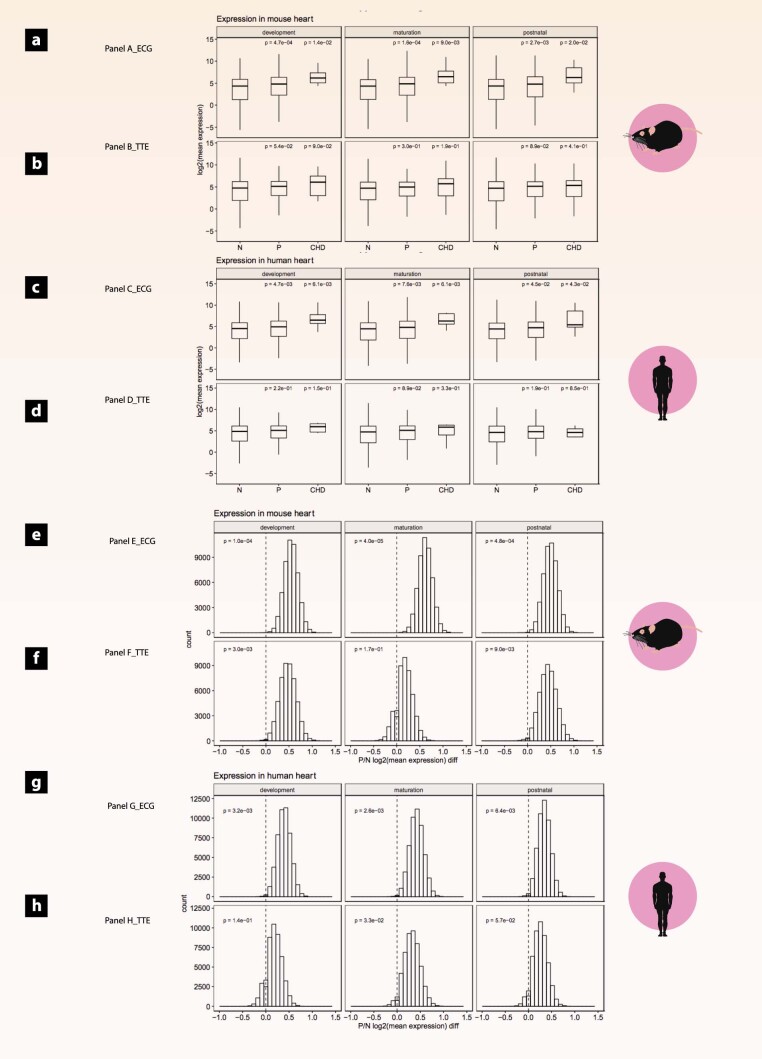
Expression profiles of mouse and human hearts in positive genes (P), CHD-associated genes (CHD) and negative genes (N, reference group). Gene based expression levels in mouse and human heart tissues performed to evaluate the impact of the size difference between positive and negative gene groups. Expression levels in known CHD genes added at all three stages for reference. Known CHD genes, however, showed significantly increased expression levels compared with positive mouse genes at all three stages. This analysis was further expanded to human heart tissue for genes with a mouse-human orthologue and presented a similar picture. Mean expression levels per groups compared using a Wilcoxon rank-sum test. X-axis denotes the gene groups evaluated; y-axis denotes the log-transformed mean expression in heart. The analysis was stratified by procedure (ECG and TTE) and developmental stages (development, maturation and postnatal). Data shown as mean, minimum, maximum and lower/upper quartiles. **a**. Expression analysis in mouse heart for ECG genes; **b**. Expression analysis in mouse heart for TTE genes. Note: RPKM, Reads per kilobase of transcript per Million mapped reads. **c**. Expression analysis in human heart for ECG genes; **d**. Expression analysis in human heart for TTE genes. Distribution of the mean expression in mouse heart differences between P and N genes. The negative gene group (higher number of genes) randomly down sampled to generate equal-size subgroups compared to the positive group. P-values denote the probability of observing by chance higher averaged expression level in negative group compared to positive group, in 50,000 permutations. The analysis stratified by procedure (**e**. ECG and **f**. TTE) and developmental stages (development, maturation and postnatal. Distribution of the mean expression in human heart differences between P and N genes. **g**. ECG and **h**. TTE and developmental stages (development, maturation and postnatal.

To explore the regulatory elements most important for normal cardiogenesis and therefore likely to play a role in congenital heart defects, we conducted chromatin immunoprecipitation followed by sequencing (ChIP–seq) analysis (ChIP-X enrichment analysis (ChEA) 2016 dataset of publicly available ChIP–seq experiments^42^). We probed for genes associated with a significant cardiac phenotype for cardio-specific enrichment compared to the 3,189 IMPC knockout genes that had no statistically significant associated cardiac phenotype using an unbiased comprehensive analysis. Genes previously identified as associated with abnormal cardiac function in knockout mice showed strong enrichment (*P* = 0.004; odds ratio, 1.31; combined score, 7.2) for heart-specific targets of the T-box transcription factor (TBX)20 next to TBX3 and TBX5 (both not significant; Supplementary Table 2), while all 3,189 genes with no significant phenotypes did not. Interestingly, the vast majority of the identified TBX20 target genes (66 of 93, ~70%) have no previous known link to CVD or are poorly annotated genes (13 of 93, ~14%; Fig. 3c). Additional gene expression analysis in both mouse and human hearts (Extended Data Fig. 2a–h) identified higher expression levels for positive genes (mean, 62.8 reads per kb of transcript per million mapped reads (RPKM); minimum, 0.01 RPKM; maximum, 10,303.04 RPKM) than for negative genes (mean, 55.22 RPKM; minimum, 0.01 RPKM; maximum, 3,270.97 RPKM) averaged across different developmental stages, stratified by ECG or TTE (Supplementary Data 2).

### Mouse genes associated with CHD phenotypes are enriched in congenital heart defect variants in human patients

With TBX20 enrichment confirmed, we further explored the translational advantage and whether our single-gene-knockout mouse mutants can provide important information on the contribution of individual gene variants to CHD and help to prioritize variants that are highly likely to contribute to or cause CHD. Recognizing that many CHDs have a complex profile of genetic mutations, we nevertheless focused on two types of mutations in this comparison, de novo mutations and LOF variants. The latter are LOF mutations that most closely resemble a single-gene knockout, whereas de novo mutations are those that appear in an individual despite not being identified in their parents. This is where the single-gene-knockout mouse is particularly useful. Here we provide additional evidence for the utility of unknown LOF candidate genes for CHD in mice to translate basic research data to inform prioritization of inherited but undiagnosed mutations in patients with CHD. We used ortholog matching between our set of 486 unknown LOF CHD candidate genes and two large-scale sequencing studies of patients with CHD and their controls: the US Pediatric Cardiac Genomics Consortium (PCGC, https://benchtobassinet.com)^43^ and the UK 100,000 Genomes Project (100KGP, https://www.genomicsengland.co.uk/)^44,45^.

**Fig. 4:**
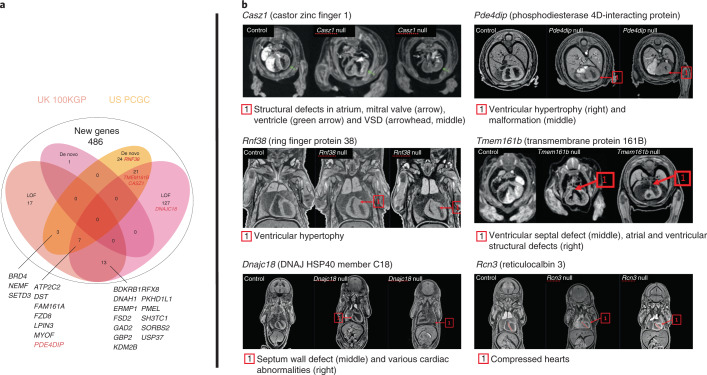
Intersection of mouse and human genes and confirmation of pathogenicity in the heart. Ortholog matching between our set of 486 non-associated LOF CHD candidate genes and those from two large-scale sequencing studies of patients with CHD and their controls: the US PCGC (https://benchtobassinet.com) and the UK 100KGP (https://www.genomicsengland.co.uk/). **a**, Intersection analysis with two CHD cohorts, PCGC and 100KGP data revealed that unknown genes from the mouse study emerged in patients with CHD with de novo and LOF variants. Most interestingly, these genes were previously classified as VUR. **b**, Confirmation of pathogenicity at induction of gene loss in *Pde4dip*-, *Casz1*-, *Rnf38*-, *Tmem161b*-, *Dnajc18*- and *Rcn3*-mutant embryos. These embryonal data show various structural abnormalities of the heart developed during cardiogenesis in knockout mice and confirm causality at gene loss.

Focusing on the 486 genes that our data and analysis designate as unassociated with CVD, alignment showed that 213 of them (44%) overlapped with human de novo and/or LOF gene alleles. More precisely, 40 genes with LOF mutations and one gene with a de novo mutation (*UST*) were aligned to data in the 100KGP, whereas 168 genes with LOF mutations and 55 genes with de novo mutations were found in the PCGC dataset (Fig. 4a). Because of their rarity, detection of de novo LOF mutations can be a powerful way to discover unappreciated CHD risk genes^1^. Here we present five representative genes causative of structural heart abnormalities in different stages of embryo cardiac development (*Casz1*, *Dnajc18*, *Pde4dip*, *Rnf38* and *Tmem161b*; Fig. 4b). The gene *PDE4DIP*^46^, encoding a scaffold protein, and *CASZ1*, a gene that encodes a zinc finger transcription factor essential for normal cardiovascular morphogenesis^47^, have been identified as both a de novo and an LOF variant in human studies and could be described as ‘de novo LOF mutations’. Heterozygous *Pde4dip*-mutant mice had decreased ventricular inner dimensions (LVIDd and LVIDs) with increased fractional shortening but normal ECG, whereas homozygous LOF deletion of *Pde4dip* caused early lethality, attributed to ventricular hypertrophy. *Casz1*-heterozygous mice had shortened QTc and ST intervals, indicating abbreviated ventricular conduction time, whereas homozygous loss of *Casz1* caused structural defects in the heart atrium, the mitral valve and the heart ventricle, VSD and early embryo lethality. The *RNF38* (encoding ring finger protein 38) gene is poorly characterized and not known to be associated with human disease. However, we were able to detect prolonged QRS, ST and QTc intervals in heterozygous *Rnf38* mutants, potentially indicating ventricular conduction delay and abnormal depolarization, while homozygous loss of *Rnf38* resulted in ventricular hypertrophy and early embryo lethality. The *TMEM161B* (encoding transmembrane protein 161B) gene has no hitherto direct experimental evidence for involvement in CHD except for preliminary data of a locus including *TMEM161B* identified in a cognitively impaired patient with comorbidities that included VSD^48^. In mice, heterozygous *Tmem161b* mutants had increased stroke volume and cardiac output that was more severe in females, whereas homozygous loss of *Tmem161b* caused manifold structural malformations affecting the vena cava, atria, ventricles and the mitral valve in addition to VSD. Another poorly characterized gene, *DNAJC18* (DNAJ heat-shock protein family (HSP40) member C18), encodes a type III HSP40–DNAJ protein family member that is involved in endoplasmic reticular protein transfer and degradation^49^. Endoplasmic reticulum stress-associated pathways such as the unfolded protein response pathway play an important role in cardiac pathophysiology^50^. However, heart abnormalities in the absence of *DNAJC18* have not been reported. Here, heterozygous *Dnajc18*-mutant mice had enlarged LVIDd with enhanced stroke volume and cardiac output, indicating slight LV dilation, while homozygous deletion (*Dnajc18*^−/−^) caused septal wall defects, severe structural cardiac malformations and early embryo death.

Notably, *RCN3* (encoding reticulocalbin 3) is a poorly characterized gene preliminary associated with lung cancer^35,51^. However, heterozygous *Rcn3*-mutant mice had impaired LV inner dimensions (LVIDd and LVIDs) with increased LVPWs, while homozygous deletion of *Rcn3* caused immediate death at birth from compressed hearts and respiratory distress. This gene was not identified among the genes carrying de novo or LOF mutations in the 100KGP and PCGC studies, but structural defects in homozygous and heterozygous mutants were compelling, indicating that this may be a previously undiscovered CHD candidate gene.

### UK Biobank highlights potential mouse-to-human translational targets

We further aimed to identify genes with a role in healthy and abnormal cardiac function in mice that could inform about human cardiology and CVD. We used the UK Biobank and matched our genotype–phenotype information from ECG and TTE. Matching with structural heart defects is not applicable in this database because it is inclusive of healthy individuals.

Association testing for ECG parameters was performed based on extracted data from precomputed summary statistics of the pan-ancestry UK Biobank dataset (from the Neale laboratory, http://www.nealelab.is/data) for three phenotypes: ventricular rate (a surrogate of the heart rate), P duration and QRS duration (UK Biobank phenotype codes 12336, 12338 and 12340, respectively). For all genes, we assessed both strict boundaries of each gene (genome build 37) and a longer sequence of the same gene ±500 kb. The greatest significance was observed in variants of *ARHGAP24*, *SMARCB1*, *ASPA*, *FAM53B* and *ABHD17B* (Supplementary Table 3a). Further analysis of the gene sequence alone identified variants in *SETBP1* (for example, 18:42,424,594[C:T]; *P* = 6.393 × 10^−17^) and *VTI1A* (for example, 10:114,505,465[G:A]; *P* = 1.770 × 10^−13^) that had significant associations with QRS duration. Eight additional genes showed suggestive *P* values (defined as *P* < 5 × 10^−6^ and *P* > 5 × 10^−8^) across all three phenotypes. Analysis of the extended sequence (gene ± 500 kb) increased the number of genes showing suggestive associations to 42, and variants in or near two additional genes showed genome-wide significant associations with QRS duration: *PRAG1* (for example, 8:8,501,230[A:G]; *P* = 7.572 × 10^−9^) and *PCYOX1L* (for example, 5:149,165,478[C:CT]; *P* = 3.479 × 10^−8^) (Supplementary Table 3b). Interestingly, we have mouse-to-human phenotypes, even for single phenotypes, that are identical, for example, *Cd300e* and *CD300E* (heart ventricular rate) or *Fndc3b* and *FNDC3B* (QRS interval length). Other genes have an associated ECG phenotype but not the same one in mice and humans, for example, *Tyms* (QRS) and *TYMS* (ventricular rate), and a few genes have an associated TTE phenotype in mice and an associated ECG phenotype in humans (Supplementary Table 3c).

Association tests for TTE parameters were carried out based on LV end-diastolic and systolic volumes (LVedv, LVesv), LV stroke volume (SV), SV index (SV adjusted for body surface area), cardiac output and cardiac index. In addition, three additional non-cardiac parameters were included such as body surface area, age and sex for genomes from the UK Biobank database with a minimum of five of the six selected TTE parameters. Five nonsynonymous coding variants were associated with a significance threshold of *P* < 1 × 10^−5^ (Supplementary Table 3d), three of which are 3′ and 5′ untranslated region variants for *DNAJC18* (Supplementary Table 3e). To assess noncoding consequences, we performed an overlap of qualifying variants associated with significant expression quantitative trait loci (eQTL) for the genes of interest at *P* < 1 × 10^−5^ for each magnetic resonance imaging (MRI) feature in each tissue from the GTEx expression database (see Methods for details). We found overlap for 50 different gene–trait pairs. The eQTL data confirmed suggestive evidence of an association between *DNAJC18* LOF and increased LV end-systolic volume and decreased systolic volume index. An additional mouse–human alignment with LV image-derived phenotypes from UK Biobank^52^ confirmed this association between *DNAJC18* LOF and LV systolic function. This analysis also revealed same-genome-wide associations with LV end-systolic–diastolic volume for five genes (*FNDC3B*, *SMARCB1*, *HILPDA*, *ALPK3* and *FBLIM1*) and an association between *CDSN* loss and increased stroke volume. Interestingly, we have mouse-to-human phenotypes, even for single phenotypes, that are identical, for example, in *Alpk3* and *ALPK3* (LVIDd, LVIDs, LVedv and LVesv) or in *Hilpda* and *HILPDA* (LVIDs and LVesv). *Alpk3* is of particular interest because this gene is involved in cardiomyocyte differentiation and is associated with several forms of cardiomyopathy^17^. Some mouse genes, however, have ECG data but no TTE data, and thus phenotypes cannot be the same in mouse–human comparisons (Supplementary Table 3f).

### Study limitations

We generated and analyzed 3,984 IMPC single-gene-knockout mouse lines on a C57BL/6N inbred background. The study focus is to access, through systematic genotyping–phenotyping in mice, a landscape of previously unknown or poorly described genes playing a role in CHD with great translational potential. We have data on so-called proof-of-concept genes but by no means data on all known cardiac genes such as *Ttn*^53^ and *Scn5a*^54^. Analysis and description of the complete data quantity was beyond the feasible scope of the presented work. However, all production–genotype data and phenotyping results (raw and statistically analyzed) are freely available (http://ftp.ebi.ac.uk/pub/databases/impc/all-data-releases). Intersection of human and mouse genes was limited primarily due to the lack of CHD gene saturation in both reviewed human studies, the mouse data we generated and our selection of LOF and de novo variants.

## Discussion

Identification of the genes, mutations and their mechanisms of action that cause and/or contribute to human inherited or de novo CHD is far from complete. We used in vivo ECG and echocardiography of young adult mice and high-resolution micro-CT imaging of embryos to identify 705 single-gene-knockout mouse lines with cardiac abnormalities. Our analysis of this gene set suggests that 75% of the genes have not been previously identified as genes associated with CHD and confirms that 25% are known cardiac genes, an important validation of our methodology to screen for CHD phenotypes and a requisite step forward in identifying genes that warrant further investigation for their role in human CHD.

We identified 297 genes that, until now, were not connected to electrical conduction disorders in the research and clinical literature. Single-gene mutation of each of these genes in knockout mouse lines resulted in significantly abnormal electrical conduction periods (that is, altered ECG intervals). Examples include *Gatm*, *Cap2* and *Elmod1*, which had a range of ECG abnormalities. The product of *GATM* is known to facilitate creatine synthesis; therefore, it is probable that abnormal creatine synthesis in cardiomyocytes impairs the dynamic requirements for cardiac excitation conduction^55^ in homozygous *Gatm*-knockout mice. The abnormal depolarization pattern observed in heterozygous *Cap2*-mutant mice may be reflective of an impairment in the contractility of cardiac muscle cells^56^. By contrast, *Elmod1* is a poorly characterized gene, only recently associated with hair cell stereocilia dysmorphology and deafness in mice^12^. Mutations in the lectin complement pathway (for example, *MASP1*) cause the rare autosomal recessive Carnevale, Mingarelli, Malpuech and Michels syndromes (3MC) that have a spectrum of developmental phenotypes but rarely include cardiac anomalies^57^. Homozygous *Masp1*-knockout mice, however, were viable and showed ventricular conduction delay with abnormal depolarization. Adding *Elmod1* and *Masp1* to the list of genes that are known to influence the heart’s electrical conduction provide unappreciated candidates for investigation.

A comprehensive assessment of all 297 genes that we identified with abnormal ECG in mutant mice is beyond the scope this study, but, given the examples above, it is very likely that this set of 297 genes represents a important resource of unknown genes involved in the cardiac conduction system, some of which may have translational potential for clinical diagnosis and therapeutic approach. These genes may be shown to have direct impact on the action potential or an impact on the genetic signaling pathways involving these genes in the development of cardiac conduction system diseases, such as sinus or atrioventricular node dysfunction or altered sub-Hisian conduction.

Our cardiac phenotyping screen also identified 297 single-gene-deletion mouse lines with abnormal dimensions and/or function of the LV myocardium (that is, altered TTE parameters). None of these genes have published links to hypertrophic and/or dilated cardiomyopathies. For example, *Fzd8*- and *Leprotl1*-mutant mice had distinct TTE features of hypertrophic cardiomyopathy. The known contribution of *Fzd8* to canonical β-catenin signaling and the WNT pathway^15^ is a likely mechanism to explore and potentially assign to the variant *Fzd8*. By contrast, the hypertrophic cardiomyopathy that we identified in *Leprotl1*-mutant mice suggests functions of this gene in addition to its known role in growth (that is, GH in the liver) and metabolism^16^. Many genes in this set are very poorly understood, yet single-gene mutation in mice and focused cardiac phenotyping identified abnormalities consistent with cardiomyopathy. Deletion of *Ints6l* or *Abhd3*, for example, resulted in dilated LV cardiomyopathy with decreased systolic function. *Ints6l* is a gene with limited information, almost no functional annotation and no known disease association before our identification of a heart phenotype consistent with cardiomyopathy. The product of *ABHD3* is known to play a multi-faceted role in the catabolism of medium-chain phospholipids, previously confirmed in an *Abhd3*-knockout mouse model with elevated myristoyl (C14)-phospholipids, including the bioactive lipid C14-lysophosphatidylcholine^58^. Here, we provide evidence for the mode of action of *Abhd3* in the myocardium. Conduction system deterioration in young adult heterozygous *Smo*-mutant mice was also intriguing. *Smo* is known to encode a G protein-coupled receptor that interacts with the patched protein, a receptor for Hedgehog proteins and a fundamental actor in embryonic development and postnatal tissue homeostasis^59^. *Smo* is also a gene that has been long debated in the context of heart disorders, particularly for its relevance in the Hedgehog signaling pathway and thus cardiac disorders. Our in vivo data show that diminished expression of *Smo* severely affects the electrical conduction system with prolonged signal from sinus node to the ventricles in structurally normal hearts, a previously unidentified association with a monogenic form of primary conduction disease that may lead to an expansion of gene function. Interestingly, homozygous E9.5 *Smo*-knockout mice died at or around E9.5 from multiple abnormalities that included craniofacial defects and malformation of ventricles and atria (Supplementary Fig. 1). These multiple abnormalities strongly suggest that the product of *Smo* has essential and pleiotropic roles in organogenesis and normal cardiac conduction system development during early embryonic development. Surprisingly, we found no link between cardiac disease and *RCN3* in human studies. Its mouse ortholog, however, showed a very compelling association with LV dilatation in heterozygous null adult mice and with compressed hearts with lethal respiratory distress in homozygous neonates. Reticulocalbin 3 is encoded by a poorly characterized gene with an unvalidated association with lung cancer^35,51^. From our work in mice, *Rcn3* is a previously undiscovered cardiac gene with an important role in cardiac development and myocardial remodeling. This gene should be the subject of further research and be included in other studies of the genetics of CHD. Systematic mouse phenotyping can complement the human data and identify genes not previously associated with CVD. Embryonic heart diagnostics of mouse embryos from knockout mouse lines that were embryo lethal or neonate subviable in particular provide a valuable resource for discovering genes causal to structural heart defects.

This approach enabled identification of cardiac phenotypes in lethal and subviable ‘essential’ genes with no previous association with normal heart development or CHD. We identified a variety of structural heart defects, with the most highly prevalent being VSD, a congenital abnormality affecting the wall dividing the left and right ventricles^5^. We confirmed that mutations in mouse genes that resulted in VSD are networked with known transcription factors (for example, NKX2-5 or TBX20 (refs. ^60,61^)), chromatin-modifying genes regulated by processes essential for cardiogenesis (for example, *Hdac1* and *Smarcb1* (ref. ^62^)) and functional pathways (for example, smoothened and WNT signaling^63^). The connectivity of mouse genes associated with the VSD phenotype with key components of cardiogenesis offered a plausible rationale for the cardiac anomalies observed in this study. As an example, homozygous *Gnao1*-knockout mice produced by the IMPC had VSD, although a different allele of *Gnao1* previously showed its involvement in the regulation of Ca^2+^ channels in the heart with no VSD^64^. VSD in *Gnao1*-null mutants was not previously reported. In humans, *GNAO1* (encoding guanine nucleotide-binding protein G(o) subunit α) is associated with infantile epileptic encephalopathy^35^, but our mouse data suggest that *GNAO1* is an unappreciated candidate gene involved in VSD in humans.

Analyses of regulatory networks enriched across our entire set of mouse genes with cardiac phenotype(s) showed strong enrichment of TBX20 targets among genes previously identified in knockouts with an impaired electrical conduction system or dysmorphic myocardium. TBX20 target enrichment added unknown functional evidence to support association of our gene set with abnormal cardiac structure phenotypes but no prior association with heart abnormalities or CHD. TBX20 is one of the critical regulators of heart development across different species^65–67^ and is associated with human CHD and adult cardiomyopathies^68–71^. Intersectional analysis of the 486 mouse genes and their human orthologs showed that the majority are previously unknown heart genes. We showed overlap between our genes and those of patients with CHD from the US PCGC^43^ and the UK 100KGP^44,45^. From this data-intersection analysis, we selected five genes with clinical human variants of unknown relevance (VUR) and showed that mutations in *Casz1* (zinc finger protein castor homolog 1), *Dnajc18* (DNAJ homolog subfamily C member 18), *Pde4dip* (myomegalin), *Rnf38* (E3 ubiquitin-protein ligase RNF38) and *Tmem161b* (transmembrane protein 161B) are pathogenic in the mouse heart. Deletion of all five genes caused severe structural defects in the heart that resulted in the death of mutant embryos. This experimental body of evidence suggests that these genes should be prioritized as pathogenic candidates in human sequencing studies.

In the UK Biobank, although biased for selection of older adults (40–69 years) and ‘healthy volunteers’ (ref. ^72^), we also found evidence of association between homologs of 31 genes from TTE and 42 of our genes of interest from ECG and MRI features of cardiac function. This particularly highlights these genes as potential targets for mouse-to-human translation and warrants further investigation into their role in healthy heart function and cardiac disease. One association that we highlighted was the association between LOF of *DNAJC18*, a gene classified as VUR in the PCGC and the UK 100KGP, and increased LV end-systolic volume and decreased systolic volume index. Likewise, the association of *SIRT1* with alterations in QRS interval length in the UK Biobank matches the results of the enrichment analysis shown in ‘Hairball’, where *SIRT1* was directly associated with heart diseases alongside 11 other genes from our study. Overall, genes associated with TTE phenotypes in our dataset are associated with the same phenotypes in the UK Biobank and therefore strengthen our findings from the IMPC mouse models. We noted that the overlap analysis with human association data was performed only for single studies. With the release of large-scale whole-exome-sequencing data, such as UK Biobank whole-exome sequencing, gene-based datasets for rare variant analysis are beginning to emerge. However, these datasets are not yet easy to query (https://genebass.org/^73^), which prevents efficient analysis. Enabling programmatic access is a key driver of large dataset adoption and should be a priority for large biobank analysts.

To date, over 500 human genes have been linked to CHD, including those encoding transcription factors, cell signaling molecules and structural proteins important for heart development. The large number of gene variants identified thus far clearly shows that diverse mechanisms lead to congenital heart defects^6^. Nevertheless, many variants are still considered to be of unknown relevance (for example, VUR), and de novo variants are emerging. Inherited cardiac disease is a generic term covering a wide variety of relatively rare diseases of the heart, mainly divided into disorders that are primarily arrhythmogenic with no obvious changes in structure and diseases that are associated with structural change, such as dilated or hypertrophic cardiomyopathy^74^. Knowledge of genetic contributions to congenital arrhythmias has made many advances, especially in heritable monogenic diseases (for example, long QT); however, the genes causing or contributing to a substantial proportion of congenital monogenic cardiac conditions have yet to be discovered^75^.

**Fig. 5:**
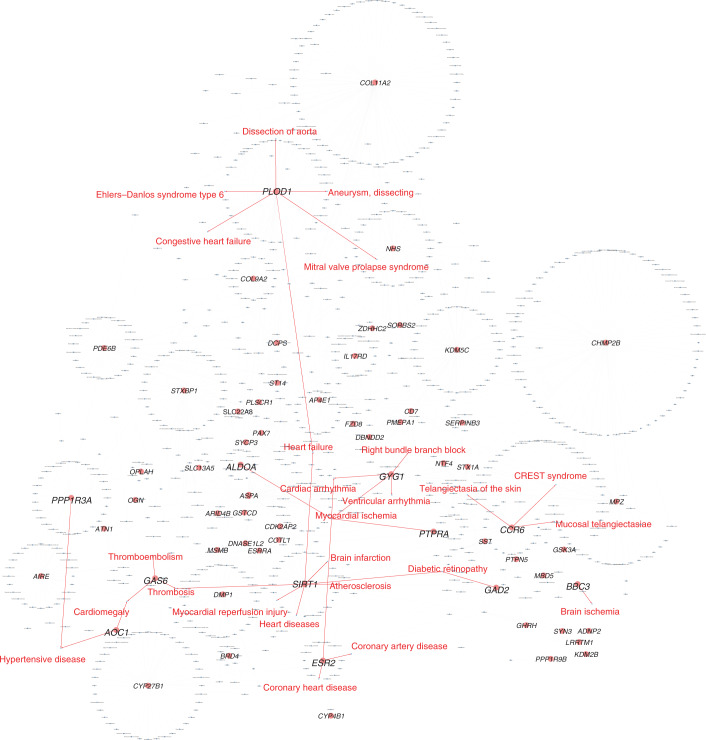
A bird’s-eye view of the gene–disease association network. CVD has diverse manifestation. To explore whether our unknown gene candidates also have potential associations with CVD other than CHD, we performed gene–disease enrichment analysis. We performed gene–disease network enrichment analysis for orthologous human–mouse cardiac knockout genes using the automated tool NetworkAnalyst^100^. The gene–disease association network was generated only with the 486 genes that have not yet been associated with cardiac dysfunction in humans. In total, 64 of 486 ‘unknown’ genes were associated with 890 diseases in the enrichment analysis ‘Hairball’ network. Twelve genes were directly related to 25 CVDs, suggesting that they may play a role in human CVDs. Nodes (light red circles), genes from the list of 486 unknown genes; light red circles with larger-font letters, genes associated with CVDs; blue rectangles, diseases; edges colors (red arrows), association of genes with CVDs; light gray arrows, associations of genes with other diseases.

We believe that the set of 705 genes included in this work is fundamentally useful to the cardiovascular community by identifying a substantial number of human–mouse orthologous genes that, when individually mutated (one mutation per mouse line), result in (but not necessarily cause) a cardiovascular phenotype. The subset of 486 genes with no previously reported association or role in CHD or more yet in CVD (Fig. 5), regardless of allele type, provides an unreported gene set of potential interest to the cardiovascular community. Additionally, LOF is by far the best-studied mechanism in human patients with CHD with and without developmental disabilities. Currently available datasets from the field of human genetics have helped uncover several genes with heterozygous de novo LOF variants, which to date have shown the strongest association and effect size with syndromic and nonsyndromic CHD. Nevertheless, estimates of these studies in the field^69,76^ can only explain about 10–40% of cases, with a higher proportion in those with neurodevelopmental disorders. Although some of these studies have examined biallelic variants, current sample sizes are often too small to detect reliable associations. Detailed phenotypic studies are needed to further unravel the underlying mechanism and reveal milder and stronger phenotypic associations depending on the background allele. These further studies on genes identified here will substantially augment the usefulness of this resource to uncover VUR and unappreciated CHD genes. Therefore, these mice are a starting point for the cardiovascular community to validate human findings and expand detailed downstream molecular investigation of genes available to the community to promote curative strategies.

## Methods

### Ethics approval and consent to participate

All IMPC institutes that breed mice and collect phenotyping data are guided by their individual ethical review, licensing and accrediting bodies, reflecting the national legislation under which they operate. Cardiovascular mouse phenotyping was carried out under the auspice of the following animal protocols: Baylor College of Medicine (AN-5896), the German Mouse Clinic Helmholtz Zentrum München (144-10 and 15-168), the Institut Clinique de la Souris Mouse Clinical Institute (4789-2016040511578546v2), Medical Research Council Harwell (97/3384), Nanjing University (NRCMM9), the Rikagaku Kenkyūjo Tsukuba Institute (Exp11-011, 12-011, 13-011, 14-009, 14-017, 15-009 and 16-008), the Centre for Phenogenomics (0153, 0275, 0277 and 0279), Jackson Laboratory (11005) and the University of California Davis (20863).

### The International Mouse Phenotyping Consortium

The IMPC is a collaborative, inter-institutional research initiative of 12 major research centers across Europe, North America and Asia^77–79^ that aims to generate and phenotype a null mutant for every protein-coding gene in the mouse genome (https://www.mousephenotype.org/)^51,77,80^. This IMPC program involves the systematic and standardized characterization of more than 20,000 knockout mice strains carried out under the uniform operating procedures detailed in the International Mouse Phenotyping Resource of Standardized Screens (IMPReSS), which was developed and validated during the pilot programs EUMORPHIA and EUMODIC^81,82^.

### IMPC gene nomination

Each IMPC center selects its genes freely; some make selections according to the preferences of funders, and others select based on scientific interest, a colocated receptor community and center-specific special focus. To this end, some centers have an organized network of experts to nominate genes of particular interest; other centers chose clones that were available when only targeted embryonic stem cells were available during the initial phase of the IMPC. The only rule is to avoid duplication and therefore not nominate genes more than once. Genes that are already known and described in their full function and disease association are of less interest. The IMPC mission focuses on characterizing many of the poorly understood genes (the ‘ignorome’) that have little to no information and publication. This strategy has made substantial contributions to our understanding of mammalian gene function in terms of sexual dimorphism^83^, pleiotropy^77^ and disease^79,84,85^.

The breadth of original data identifies previously uncharacterized disease genes in the mouse and additional phenotypes for genes with existing mutant lines mimicking the associated disorder.

There is also a feature on the IMPC website (https://www.mousephenotype.org) with which the public community can express interest in specific genes and actively participate in the nomination process.

### IMPC data collection

The IMPC has 21 international phenotyping centers that use high-throughput pipelines to phenotype knockout mouse strains. These centers are generating homozygous knockout mouse strains for every protein-coding gene in the mouse genome, producing seven males and seven females per gene.

After weaning, viability of offspring was monitored. When homozygous mice were viable, the animals were phenotyped following the procedures as indicated in the early adult (EA) pipeline. When homozygous mice were not viable, additional homozygous mice entered the embryo pipeline and the heterozygous mice, when viable, were phenotyped following the EA pipeline. For sample sizes, 14 homozygous knockout mice (seven female and seven male mice) were phenotyped for each gene. Wild-type mice were continually tested using the same protocols to provide baseline ‘normal’ values.

The phenotyping pipeline generated multiple types of data. Phenotype parameters were continuous data (for example, cholesterol levels) or qualitative values (for example, presence or absence of cataracts). Additionally, images (for example, X-rays, echocardiograms, LacZ staining, histology) were collected as part of the protocols. Statistical analysis was performed to identify outlier values. These outliers were then annotated with terms from the mouse phenotype ontology, for example, high circulating cholesterol. This text contained data from the EA pipeline. This pipeline contained a wide range of in vivo phenotyping procedures carried out from 9 to 15 weeks with body weights measured weekly from 4 to 16 weeks (detailed information on the ‘IMPC EA pipeline’ is provided at https://www.mousephenotype.org/impress/PipelineInfo).

Cardiovascular data were gathered at week 12 with ECG and/or echocardiography. Some procedures were mandatory for all centers, assuring key data were provided (see https://www.mousephenotype.org/impress/index for high-level detail).

At the time when this study was carried out, the following centers provided ECG and TTE data: Baylor College of Medicine, the Helmholtz Centre Munich, MRC Harwell and the Institut Clinique de la Souris. ECG data were provided by Jackson Laboratory, the RIKEN Tsukuba Institute BioResource Center, the Toronto Centre for Phenogenomics and the University of California, Davis. In addition, TTE data were provided by Nanjing University. These centers were not yet involved in the data release (DR)10.1 data collection for this publication: the Wellcome Trust Sanger Institute, the Czech Centre for Phenogenomics and the Korea Mouse Phenotyping Center.

### Mouse phenotyping

We used data collected from the IMPC phenotyping pipeline including cardiovascular screening, carried out at 12 weeks of age, to determine cardiovascular health using two different procedures, namely, high-throughput electrocardiogram recordings of conscious mice if not stated differently (IMPC_ECG_001 and IMPC_ECG_002) and TTE recordings of anesthetized mice if not stated differently (IMPC_ECH_001) to assess the morphology and functionality of the heart.

### Electrocardiography recording

As even modest handling of mice may induce alterations in heart rate^86^, each mouse was permitted to acclimatize on the ECG recording platform for 10 min before measurement. Furthermore, cage mates were placed on the adjacent platform unit to provide company. Electrocardiograms were recorded in a dim and quiet analysis room. To eliminate circadian influences, electrocardiograms were recorded at the same time of day. A disposable lead plate (Mouse Specifics) was embedded in the floor of the platform and spaced to provide contact between the electrodes and animals’ paws, providing an ECG signal equivalent to that of Einthoven lead II. Only runs in which at least 15 ECG beats could be included in the analysis were chosen. Data were analyzed using standard protocols for ECG signal analysis by eMouse (Mouse Specifics). The software uses a peak-detection algorithm to find the peak of R waves and to calculate heart rate. The software plots its interpretation of P, Q, R, S and T for each beat so that heart rate, QRS duration, PQ interval, PR interval, QT interval and ST interval are measured and reported automatically. In addition, each trace was examined for clear P, Q, R, S and T peaks before accepting the automatic calculations. Sensitivity was corrected manually in case R peaks were not chosen correctly. Noise and motion artifacts are rejected automatically by the software. As in mice the T wave often merges with the final part of the QRS complex^87^, the software automatically defines the end of the T wave of each signal as the point where the signal intersects the isoelectric line. HRV was calculated as the mean of the differences between sequential heart rates for the complete set of ECG signals. QT intervals were rate corrected (QTc) by applying the equation recommended by Mitchell et al.^88^. Detailed experimental protocols for the IMPC phenotyping procedures are available for general access at https://www.mousephenotype.org/IMPReSS.

### Transthoracic echocardiography recording

Body weights were taken shortly before TTE. For TTE recordings of anesthetized mice, the animal was placed in an induction chamber and anesthetized with gaseous anesthetic. Once the animal was sedated, it was moved to a nose cone for hair removal in the area of measurement. Once the hair was removed, the animal was moved to the imaging platform, its paws were taped to the ECG lead plates, and a rectal probe was inserted to monitor body temperature, which was maintained at 36–37 °C. During imaging, anesthesia was adjusted to maintain proper heart rate and prevent the animal from waking up. For TTE examination of awake animals, mice were firmly held by the nape of the neck (in the supine position) in the palm of one hand with the tail held tightly between the last two fingers. Prewarmed ultrasound gel was placed on the chest at the area of imaging, and TTE recordings started in short-axis mode with papillary muscles being the point of reference. Images were obtained in parasternal long-axis B mode and short-axis views in M mode with at least three images per mode. Once imaging was complete, the animal was removed from the platform and allowed to recover on top of a heating pad.

Qualitative and quantitative measurements were made offline using analytical software (VisualSonics). LVIDs, LVIDd, systolic and diastolic interventricular septum thickness (IVSs and IVSd), LVPWs and LVPWd were measured in three consecutive beats according to the American Society of Echocardiography leading-edge method^89^ as a measure of the actual visualized thickness of the ventricular septum and other chamber dimensions as defined by the actual tissue–blood interface. Papillary muscles should be excluded from the cavity in the tracing. Fractional shortening was calculated as percent fractional shortening = ((LVIDd − LVIDs)LVIDd^−1^) × 100. Ejection fraction was calculated as percent ejection fraction = 100 × ((LVvold − LVvols)LVvold^−1^) with LVvol = ((7.0(2.4 + LVID)^−1^ × LVID^3^), where LVvold is end-diastolic volume and LVvols is end-systolic volume.

The corrected LV mass was calculated as 0.8(1.053 × ((LVIDd + LVPWd + IVSd)^3^ − LVIDd^3^)). SV is the volume of blood pumped from one ventricle of the heart with each beat. The stroke volume of the left ventricle was obtained by subtracting LVvols from LVvold. Heart rate was determined from the cardiac cycles recorded on the M-mode tracing, using at least three consecutive systolic intervals. In addition, respiratory rate was calculated by measuring three consecutive respiratory intervals.

Importantly, each center records relevant metadata parameters including equipment manufacturer, equipment model, recording environment, anesthetic agent and anesthetic dose.

### Statistical methods

The workflow can be described in two major stages: (1) data collection (in Supplementary Methods) and (2) statistical analysis.

The statistical analysis complies with the IMPC statistical pipeline and implementation in R^90^ (version 3.4.0) and applies the R packages SmoothWin^91^ and PhenStat^92–94^. Outcomes of the statistical pipeline, including 57,500+ analyses and results, are then assigned a mammalian phenotype term using the standard definitions in IMPReSS (https://www.mousephenotype.org/impress/index). In brief, the Mammalian Phenotype Ontology is a community effort to provide standard terms for annotating phenotypic data (http://www.informatics.jax.org/vocab/mp_ontology). This browser can be used to view terms, definitions and term relationships in a hierarchical display (http://www.informatics.jax.org/userhelp/VOCAB_mp_browser_help.shtml).

Statistical results as well as the raw data and the assigned mammalian phenotype terms are available at https://zenodo.org. Windowed and standard (non-windowed^79^) analyses for the IMPC data remain comparable on this web interface (https://zenodo.org).

### Human and mouse orthologs

To obtain human orthologs for mouse genes, we applied the protein-coding file from the HGNC website (20190617) and the HCOP file (human-to-mouse orthologs 20190617) (fixed for 12 unique inference methods; that is, methods that appear multiple times were counted only once). We considered good-quality orthologs as ‘protCod_score ≥ 5_max’ and defined ortholog-inference score ≥5 (that is, the inference is supported by five or more methods); both directions (mouse-to-human and human-to-mouse) with ‘max’ score (filtering out ‘dup_max’ and ‘no_max’ scores) had a total of 16,882 inferences, included one-to-one and one-to-many orthologs.

### IMPC embryonic data

Knockout mouse strains differ in viability, with up to one-quarter of mutations being lethal. Each IMPC gene-knockout strain is assessed for viability by examination of litters produced from mating heterozygous animals. A mutation is declared lethal if no homozygous null pups are detected at genotyping age (P13–P17), while it is declared subviable if homozygous null pups constitute less than 12.5% of the progeny. For lethal mutations, embryos are phenotyped in the embryonic or perinatal lethal pipeline.

Preparation of mouse embryos for micro-CT imaging and phenotyping was described previously^51^. Briefly, pregnant timed mated dams were euthanized according to an approved protocol from the local Institutional Animal Care and Usage Committee (IACUC). Embryos were dissected and collected in warm 1× PBS, while yolk sacs from each embryo were collected for genotyping. Each embryo was immersed in individual sample tubes with a sufficient amount of ice-cold 4% paraformaldehyde and fixed at 4 °C (E8.5–E15.5, overnight; E18.5, 3 d). After fixation, a hydrogel-based tissue-scaffolding protocol (STABILITY) was used to prevent sample shrinkage and deformation for embryos at E15.5 and beyond^51,95,96^. To scaffold the embryo with hydrogel, each sample was transferred to a 50-ml conical tube, immersed in 20 ml STABILITY buffer and incubated at 4 °C for 3 d to allow the polymer to diffuse through the embryo. Samples were then placed in a desiccator to remove the air in the sample tubes with a bench-top vacuum for 10 min, followed by purging with nitrogen gas at 10 psi for 5 min. The cross-linking reaction was initialized by incubating samples at 37 °C for 3 h. After cross-linking, the external hydrogels were removed from the specimens, and the samples were stored in 1× PBS with 0.1% (wt/vol) sodium azide at 4 °C until ready for imaging. Iodine solution (0.1 N) was used to contrast the soft tissue of the embryo for micro-CT imaging. Samples were immersed in iodine solution for staining on a nutator at room temperature. The minimum required staining time depends on the stage and size of the embryos (E8.5–E15.5, overnight; E18.5, 3 d). Embryos were then mounted in 56-mm capped sample tubes in 1% (wt/vol) agarose immediately before imaging. The raw data for three-dimensional imaging of the samples were acquired with a SKYSCAN 1272 micro-CT scanner (Bruker) along the anterior–posterior axis, and acquired projection images were then reconstructed by NRecon Reconstruction (version 1.6.9.8, Bruker) software. Image-processing software CTVox (Bruker) and Slicer (https://www.slicer.org/) were used for rendering the reconstructed three-dimensional volume data and data analysis.

Briefly, among lethal lines, approximately 50% of animals die before E9.5, 15% are viable at E9.5 and lethal before E15.5 and the remaining 35% are usually lethal prenatally between E15.5 and birth. Among our 486 genes of interest, 321 (66%) were viable and their corresponding lines will not be subjected to embryo phenotyping and 24% were lethal. Based on previous IMPC distributions, of the 77 lethal lines, we would expect 50% to be lethal before E9.5, leaving 38 genes for which we would expect to obtain embryonic imaging data. Among these, we would expect data for six lines at E9.5 (15% of lines viable at E9.5 are then lethal at E12.5 or E15.5) and E15.5 or E18.5 data for the remaining 32 lines. For the subviable lines, we would expect data at E18.5 for 29 genes. Thus, even when starting with a large collection of lines, only a limited set would have embryo data due to operational and biological reasons.

### UK Biobank database

We extracted data for LVedv, LVesv, SV, SV index (SV adjusted for body surface area), cardiac output and cardiac index. In addition, three additional non-cardiac parameters were examined such as body surface area, age and sex for genomes of 39,624 individuals in the UK Biobank database when at least one phenotype was not missing. We averaged values over the second and third visit to minimize missingness and adjusted MRI traits for age and sex as well as LVedv and LVesv for body surface area. We restricted the analysis to complete cases only and excluded adjusted outlier values above 200, 4 and 10 for both end volumes, cardiac index and cardiac output, respectively. We performed step 1 of REGENIE 2 version 2.2 using the 20 first genetic principal components on the directly typed version 2 release of UK Biobank filtered for MAF (0.01), MAC (100), HWE (*P* > 1 × 10^−15^) and a sample and genotyping rate of 0.9. To build the set of qualifying variants for which association tests are performed, we queried the Ensembl REST API Homology endpoint and selected only human genes with a one-to-one homology status with the genes of interest. We extracted gene coordinates using the Ensembl REST API Lookup endpoint and extended them by 1 Mbp on either side. The resulting regions were merged using bedtools merge and extracted from the imputed version 2 dataset, restricted to the samples present in the phenotype file using plink version 2.00a3LM AVX2 Intel (4 August 2021). Association was carried out on the resulting 13,193,809 variants using step 2 of REGENIE version 2.2, and suggestively significant variants (log_10_ (*P* > 5)) were extracted using R data.table (Supplementary Table 3).

Exon coordinates were queried using the Ensembl API Lookup endpoint, and exonic variants were identified using bedtools intersect. The consequences of the resulting variants were assessed using the Ensembl REST API VEP endpoint. For eQTL mapping, we downloaded GTEx version 8, mapped IDs to build 37 coordinates using the ‘2017-06-05_v8’ lookup table and subset the data to only include eQTL for the genes of interest. We excluded variants for which eQTL direction was not concordant across tissues for a given gene and merged these eQTL data with the REGENIE output. We aggregated variants by gene and classified them based on whether eQTL and GWAS directions were concordant or discordant and computed the resulting proportion. For all genes considered, most variants (77% at the lowest) agreed on either concordance or discordance, meaning that a positive effect on expression clearly correlated with an increased (for concordant genes) or decreased (for discordant genes) value of the measured trait. We also report, for every gene–trait pair, the minimum association *P* value found for any variant that is also an eQTL (Supplementary Table 3).

### Reporting Summary

Further information on research design is available in the Nature Research Reporting Summary linked to this article.

### Supplementary information

Supplementary Information Supplementary Methods and Fig. 1

Reporting Summary

Supplementary Data 1 Comprehensive genotype and phenotype information of all 705 significant mouse genes: abnormal cardiac phenotypes (*q* value < 0.05) detected by ECG (left), TTE (middle) or both ECG and TTE (right) are listed per gene. Abbreviations: QTc dispersion, QT dispersion corrected for heart rate. Note that arrows indicate phenotype direction in knockout compared to C57BL/6N mice: increased, ↑; decreased, ↓. Colors indicate sex effect: both, in males; only, in females only.

Supplementary Data 2 Gene-based expression levels from mouse heart tissue: expression levels in mouse heart in RPKM are presented per gene across different developmental stages, stratified by ECG or TTE.

Supplementary Table 1 Comprehensive overview of all 3,894 IMPC null alleles (DR10.1): data include information on MGI gene (Marker-ID), Entrez gene ID, Ensembl ID, gene symbol, IMPC allele symbol, zygosity, procedure (ECG or TTE) and gene category (positive, significant phenotype; negative, no significance between mutant and controls). Associations for an additional 45 IMPC genes from our study with structural heart defects, particularly the development of VSD obtained by reading and manual annotation of experimental findings from relevant publications, primarily peer-reviewed ‘small-scale experiment’ literature. OMIM (https://www.omim.org/) and Orphanet (https://www.orpha.net/) queries confirmed 23 genes associated with pleiotropic neurodevelopmental disorders that include sporadic congenital heart defects or malformations. Comprehensive upstream literature-based analysis of all 705 genes irrespective of the test modality and type of phenotype abnormality identified 486 mouse–human orthologous genes with no previous link to CVD.

Supplementary Table 2 In silico enrichment analysis of all 3,894 IMPC mouse genes: comprehensive information on the ChEA 2016 dataset of publicly available ChIP–seq experiments^42^ to identify enriched regulatory networks across our total set of 3,894 mouse genes. Presented for the positive (significant) and nonsignificant (negative) genes. Strong enrichment was considered at combined score >5 and adjusted *P* value <0.05.

Supplementary Table 3 Comprehensive information on the UK Biobank query: association testing was carried out on the resulting 13,193,809 variants using step 2 of REGENIE version 2.2 and suggestively significant variants (log_10_ (*P* > 5)). a, Comprehensive information on ECG parameter-association testing based on extracted data from precomputed summary statistics on the pan-ancestry UK Biobank dataset. b, Extended sequence analysis (gene ± 500 kb). c, Mouse–human ECG phenotype comparison. d, Comprehensive information on TTE parameter-association testing based on extracted data from the UK Biobank dataset. e, Three of five are 3′ and 5′ untranslated region variants for *DNAJC18*. f, Mouse–human ECG phenotype comparison.

## Data Availability

All data generated or analyzed during this study are included in this published article and its Supplementary Information. IMPC data are open access for public. For single-gene search, visit https://www.mousephenotype.org/data/search. For batch queries, please visit https://www.mousephenotype.org/data/batchQuery. To download a particular DR, visit http://ftp.ebi.ac.uk/pub/databases/impc/all-data-releases/. Support for particular DR (here we used DR10.1) download can be found at https://www.mousephenotype.org/help/programmatic-data-access/.
