# Publisher Correction: Extensive identification of genes involved in congenital and structural heart disorders and cardiomyopathy

**DOI:** 10.1038/s44161-022-00072-2

**Published:** 2022-04-20

**Authors:** Nadine Spielmann, Gregor Miller, Tudor I. Oprea, Chih-Wei Hsu, Gisela Fobo, Goar Frishman, Corinna Montrone, Hamed Haseli Mashhadi, Jeremy Mason, Violeta Munoz Fuentes, Stefanie Leuchtenberger, Andreas Ruepp, Matias Wagner, Dominik S. Westphal, Cordula Wolf, Agnes Görlach, Adrián Sanz-Moreno, Yi-Li Cho, Raffaele Teperino, Stefan Brandmaier, Sapna Sharma, Isabella Rikarda Galter, Manuela A. Östereicher, Lilly Zapf, Philipp Mayer-Kuckuk, Jan Rozman, Lydia Teboul, Rosie K. A. Bunton-Stasyshyn, Heather Cater, Michelle Stewart, Skevoulla Christou, Henrik Westerberg, Amelia M. Willett, Janine M. Wotton, Willson B. Roper, Audrey E. Christiansen, Christopher S. Ward, Jason D. Heaney, Corey L. Reynolds, Jan Prochazka, Lynette Bower, David Clary, Mohammed Selloum, Ghina Bou About, Olivia Wendling, Hugues Jacobs, Sophie Leblanc, Hamid Meziane, Tania Sorg, Enrique Audain, Arthur Gilly, Nigel W. Rayner, IMPC consortium, Juan A. Aguilar-Pimentel, Lore Becker, Lillian Garrett, Sabine M. Hölter, Oana V. Amarie, Julia Calzada-Wack, Tanja Klein-Rodewald, Patricia da Silva-Buttkus, Christoph Lengger, Claudia Stoeger, Raffaele Gerlini, Birgit Rathkolb, Daniela Mayr, John Seavitt, Angelina Gaspero, Jennie R. Green, Arturo Garza, Ritu Bohat, Leeyean Wong, Melissa L. McElwee, Sowmya Kalaga, Tara L. Rasmussen, Isabel Lorenzo, Denise G. Lanza, Rodney C. Samaco, Surabi Veeraragaven, Juan J. Gallegos, Petr Kašpárek, Silvia Petrezsélyová, Ruairidh King, Sara Johnson, James Cleak, Zsombor Szkoe-Kovacs, Gemma Codner, Matthew Mackenzie, Adam Caulder, Janet Kenyon, Wendy Gardiner, Hayley Phelps, Rhys Hancock, Claire Norris, Michayla A. Moore, Audrie M. Seluke, Rachel Urban, Coleen Kane, Leslie O. Goodwin, Kevin A. Peterson, Matthew Mckay, Jenn J. Cook, Jacob P. Lowy, Michael McFarland, Joshua A. Wood, Brandon J. Willis, Heather Tolentino, Todd Tolentino, Michael Schuchbauer, Jason Salazar, Jennifer Johnson, Rebecca Munson, Abdel Ayadi, Guillaume Pavlovic, Marie-Christine Birling, Sylvie Jacquot, Dalila Ali-Hadji, Philippe Charles, Philippe Andre, Marie-France Champy, Fabrice Riet, Igor Vukobradovic, Zorana Berberovic, Dawei Qu, Ruolin Guo, Abigail D’Souza, Ziyue Huang, Susan Camilleri, Milan Ganguly, Hibret Adissu, Mohammed Eskandarian, Xueyuan Shang, Kyle Duffin, Catherine Xu, Kyle Roberton, Valerie Laurin, Qing Lan, Gillian Sleep, Amie Creighton, Lauri Lintott, Marina Gertsenstein, Monica Pereira, Sandra Tondat, Amit Patel, Maribelle Cruz, Alex Bezginov, David Miller, Wang Hy, Atsushi Yoshiki, Nobuhiko Tanaka, Masaru Tamura, Zhiwei Liu, Olga Ermakova, Anna Ferrara, Paolo Fruscoloni, Claudia Seisenberger, Antje Bürger, Florian Giesert, Genomics England Research Consortium, J. C. Ambrose, P. Arumu gam, R. Bevers, M. Bleda, F. Boardman-Pretty, C. R. Boustred, H. Brittain, M. J. Caulfield, G. C. Chan, T. Fowler, A. Giess, A. Hamblin, S. Henderson, T. J. P. Hubbard, R. Jackson, L. J. Jones, D. Kasperaviciute, M. Kayikci, A. Kousathanas, L. Lahnstein, S. E. A. Leigh, I. U. S. Leong, F. J. Lopez, F. Maleady-Crowe, M. McEntagart, F. Minneci, L. Moutsianas, M. Mueller, N. Murugaesu, A. C. Need, P. O‘Donovan, C. A. Odhams, C. Patch, D. Perez-Gil, M. B. Pereira, J. Pullinger, T. Rahim, A. Rendon, T. Rogers, K. Savage, K. Sawant, R. H. Scott, A. Siddiq, A. Sieghart, S. C. Smith, A. Sosinsky, A. Stuckey, M. Tanguy, A. L. Taylor-Tavares, E. R. A. Thomas, S. R. Thompson, A. Tucci, M. J. Welland, E. Williams, K. Witkowska, S. M. Wood, Marc-Phillip Hitz, Eleftheria Zeggini, Eckhard Wolf, Radislav Sedlacek, Steven A. Murray, Karen L. Svenson, Robert E. Braun, Jaqueline K. White, Lois Kelsey, Xiang Gao, Toshihiko Shiroishi, Ying Xu, Je Kyung Seong, Fabio Mammano, Glauco P. Tocchini-Valentini, Arthur L. Beaudet, Terrence F. Meehan, Helen Parkinson, Damian Smedley, Ann-Marie Mallon, Sara E. Wells, Harald Grallert, Wolfgang Wurst, Susan Marschall, Helmut Fuchs, Steve D. M. Brown, Ann M. Flenniken, Lauryl M. J. Nutter, Colin McKerlie, Yann Herault, K. C. Kent Lloyd, Mary E. Dickinson, Valerie Gailus-Durner, Martin Hrabe de Angelis

**Affiliations:** 1 Institute of Experimental Genetics, German Mouse Clinic, Helmholtz Center Munich (GmbH), German Research Center for Environmental Health, Neuherberg, Germany; 2 Department of Internal Medicine, Division of Translational Informatics and Center of Biomedical Research Excellence in Autophagy, Inflammation, and Metabolism, UNM Health Sciences Center and UNM Comprehensive Cancer Center, Albuquerque, NM USA; 3 Department of Rheumatology and Inflammation Research, Institute of Medicine, Sahlgrenska Academy at University of Gothenburg, Gothenburg, Sweden; 4 Novo Nordisk Foundation Center for Protein Research, Faculty of Health and Medical Sciences, University of Copenhagen, Copenhagen, Denmark; 5 Department of Molecular Physiology and Biophysics, Baylor College of Medicine, Houston, TX USA; 6 European Molecular Biology Laboratory, European Bioinformatics Institute, Wellcome Trust Genome Campus, Hinxton, UK; 7 Institut für Humangenetik, Technische Universität Munich, Munich, Germany; 8 Klinik und Poliklinik Innere Medizin I, Klinikum Rechts der Isar, Technical University of Munich, Munich, Germany; 9 Department of Congenital Heart Defects and Pediatric Cardiology, German Heart Center Munich, Technical University Munich, Munich, Germany; 10 DZHK (German Centre for Cardiovascular Research), partner site Munich Heart Alliance, Munich, Germany; 11 Experimental and Molecular Pediatric Cardiology, German Heart Center Munich, Technical University Munich, Munich, Germany; 12 DZHK (German Centre for Cardiovascular Research), partner site Munich, Munich, Germany; 13 Research Unit of Molecular Epidemiology, Institute of Epidemiology II, Helmholtz Zentrum Munich, Munich, Germany; 14 German Center for Diabetes Research (DZD), Neuherberg, Germany; 15 Czech Centre for Phenogenomics, Institute of Molecular Genetics of the Czech Academy of Sciences, Prague, Czech Republic; 16 Mammalian Genetics Unit and Mary Lyon Centre, Medical Research Council Harwell Institute, Harwell, UK; 17 The Jackson Laboratory, Bar Harbor, ME USA; 18 Mouse Biology Program, University of California, Davis, Davis, CA USA; 19 Université de Strasbourg, CNRS, INSERM, IGBMC, Institut Clinique de la Souris, PHENOMIN-ICS, Illkirch, France; 20 Department of Congenital Heart Disease and Pediatric Cardiology, University Hospital of Schleswig-Holstein, Kiel, Germany; 21 German Center for Cardiovascular Research (DZHK), Kiel, Germany; 22 Institute of Translational Genomics, Helmholtz Zentrum München, German Research Center for Environmental Health, Neuherberg, Germany; 23 Wellcome Centre for Human Genetics, Nuffield Department of Medicine, University of Oxford, Oxford, UK; 24 Oxford Centre for Diabetes, Endocrinology and Metabolism, Radcliffe Department of Medicine, University of Oxford, Oxford, UK; 25 Wellcome Sanger Institute, Wellcome Genome Campus, Hinxton, UK; 26 TUM School of Medicine, Technical University of Munich and Klinikum Rechts der Isar, Munich, Germany; 27 Institute of Molecular Animal Breeding and Biotechnology, Gene Center, Ludwig-Maximilians-University Munich, Munich, Germany; 28 The Centre for Phenogenomics, Toronto, Ontario Canada; 29 Lunenfeld-Tanenbaum Research Institute, Sinai Health System, Toronto, Ontario Canada; 30 SKL of Pharmaceutical Biotechnology and Model Animal Research Center, Collaborative Innovation Center for Genetics and Development, Nanjing Biomedical Research Institute, Nanjing University, Nanjing, China; 31 RIKEN BioResource Center, Tsukuba, Japan; 32 Cambridge-Suda Genomic Research Center, Soochow University, Suzhou, China; 33 Korea Mouse Phenotyping Consortium (KMPC) and BK21 Program for Veterinary Science, Research Institute for Veterinary Science, College of Veterinary Medicine, Seoul National University, Seoul, South Korea; 34 CNR Institute of Biochemistry and Cell Biology, Monterotondo, Rome, Italy; 35 William Harvey Research Institute, Charterhouse Square Barts and the London School of Medicine and Dentistry Queen Mary University of London, London, UK; 36 Institute of Developmental Genetics, Helmholtz Zentrum Munich, German Research Center for Environmental Health GmbH, Neuherberg, Germany; 37 Department of Developmental Genetics, TUM School of Life Sciences, Technische Universität Munich, Freising, Germany; 38 Deutsches Institut für Neurodegenerative Erkrankungen (DZNE) Site Munich, Munich, Germany; 39 Munich Cluster for Systems Neurology (SyNergy), Adolf-Butenandt-Institut, Ludwig-Maximilians-Universität Munich, Munich, Germany; 40 The Hospital for Sick Children, Toronto, Ontario Canada; 41 Université de Strasbourg, CNRS, INSERM, Institut de Génétique Biologie Moléculaire et Cellulaire, IGBMC, Illkirch, France; 42 Department of Surgery, School of Medicine, University of California, Davis, Davis, CA USA; 43 Department of Experimental Genetics, TUM School of Life Science, Technische Universität Munich, Freising, Germany; 44 Genomics England, London, UK; 45 William Harvey Research Institute, Queen Mary University of London, London, UK

**Keywords:** Genetic predisposition to disease, Cardiovascular genetics

Correction to: *Nature Cardiovascular Research* 10.1038/s44161-022-00018-8, published online 17 February 2022.

In the version of this article initially published, there were errors in Figure 1. In Fig. 1f, due to a production error, the large panels for *Ap4e1*^−/−^ mutant and control were interchanged and have been restored. In Fig. 1d, due to a submission error, large and small panels for *Leprotl1*^−/−^ mutant and control were incorrect, and the smaller Fig. 1e *Alpk3*^−/−^ control panel was also provided in error. The images have been replaced in the HTML and PDF versions of the article, and original and revised versions of Fig. 1d–f are shown below.

**Fig. 1:**
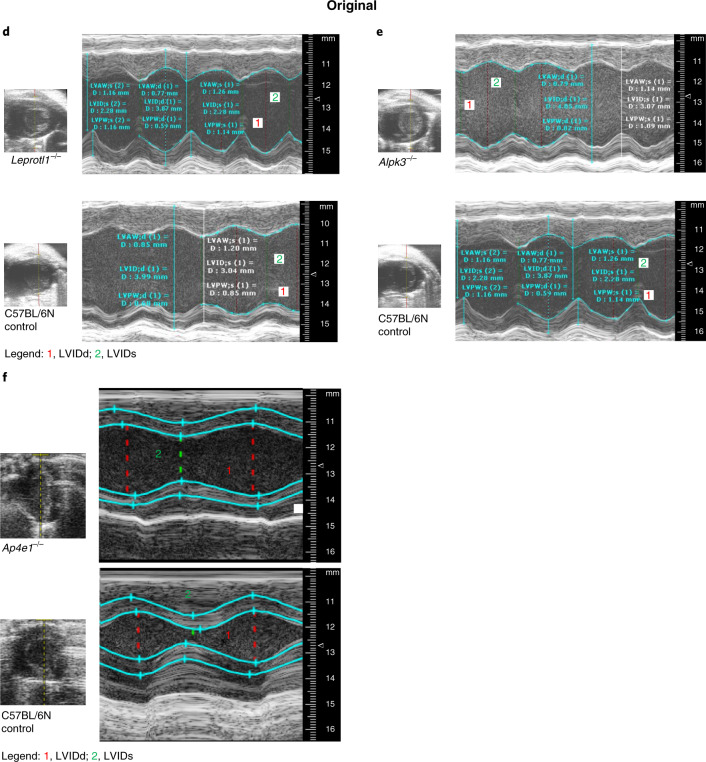
Original Fig. 1d–f.

**Fig. 1:**
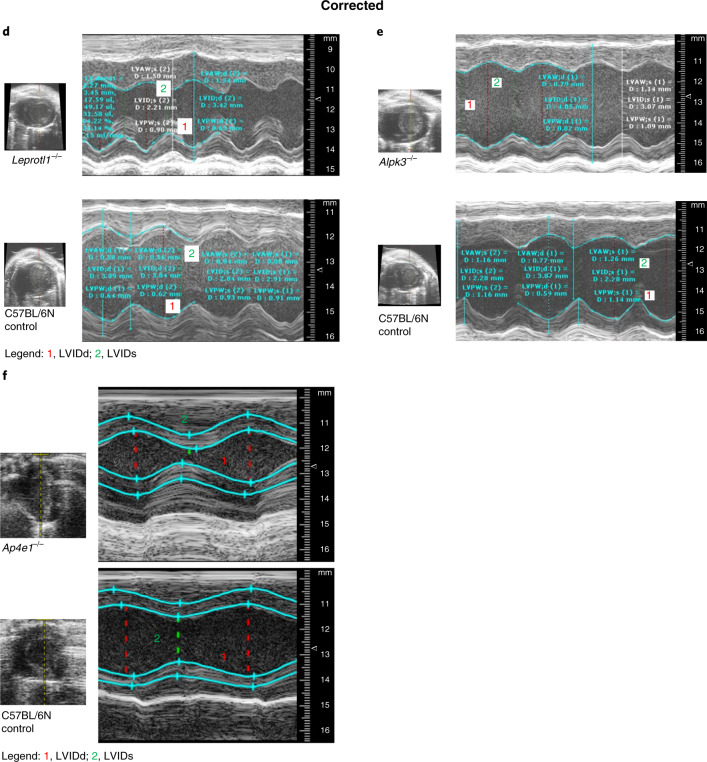
Corrected Fig. 1d–f.

