# Supplementary material for: Extensive identification of genes involved in congenital and structural heart disorders and cardiomyopathy
Source: Nat Cardiovasc Res. 2022 Feb 17;1(2):157–73. doi: 10.1038/s44161-022-00018-8 (PMC11358025; doi:10.1038/s44161-022-00018-8)

nature portfolio

https://doi.org/10.1038/s44161-022-00018-8

Supplementary information

Extensive identification of genes involved in congenital and structural heart disorders and cardiomyopathy

In the format provided by the authors and unedited

Genotyping and allele quality control

Each mutant line underwent allele validation at the center where the mouse model was produced. The consortium website (http://www.mousephenotype.org) has a webpage dedicated to each gene with a link to the allele created. A combination of short-range PCR, quantitative PCR and/or non-radioactive Southern blot was applied to validate targeted alleles[1,2]. These included allele-specific assays started at the level of the ES cell as well as of the resulting mouse. The CRISPR-engineered alleles were confirmed by PCR amplification of the targeted locus and Sanger sequencing to validate introduction of the required editing event. All QC data, including for CRISPR-engineered alleles and the guides employed, and Sanger sequencing results of the engineered allele, are deposited centrally in the iMits (International Micro-injection tracking system, https://www.mousephenotype.org/imits/) and GenTaR (https://www.gentar.org/tracker) databases. The IMPC database tracks allele generation and enables all mouse clinics to assess data for any generated strain. These data are freely available through the IMPC portal. Additionally, the full allele-specific genotyping details are communicated when mice or germplasm are distributed from an IMPC repository.

Mouse generation

Targeted ES cell clones were obtained in most cases from the European Conditional Mouse Mutagenesis Program (EUCOMM) and Knockout Mouse Project (KOMP) resource[3 4] and injected into BALB/cAnN or C57BL/6J blastocysts or aggregated with ICR morula for chimera generation[5]. The resulting chimeras were mated to C57BL/6N mice, and the progeny were screened to confirm germline transmission. In a small number of cases, CRISPR/Cas9-mediated non-homologous end joining was used to generate loss-of-function indels or exon deletions via pronuclear injection or electroporation of C57BL/6N zygotes[6]. In both instances, following recovery of germline-transmitting progeny, heterozygotes were intercrossed to produce homozygous mutants. All strains are available from http://www.mousephenotype.org/.

Mouse Phenotyping

Data was derived from postnatal mice that were phenotyped under the adult and embryonic phenotype pipeline (https://www.mousephenotype.org/impress). Briefly, this pipeline conducted 16 tests, each with a set of phenotyping procedures. Phenotyping recorded appearance, behavior, or organ function across a spectrum of organs and tissues. All IMPC phenotyping data is shared with the public through the IMPC website. Experimental procedures are detailed also under IMPRESS (International Mouse Phenotyping Resource of Standardised Screens - https://www.mousephenotype.org/impress). Although we mined the entire phenotyping data when investigating co-phenotypes, our study primarily investigated the cardiovascular phenotyping data obtained in postnatal week 12.

A minimum of 7 male and 7 female mutants were phenotyped, giving a minimum of 14 mice per line. In all experiments, mutants were matched with wild-type (wt) animals, with matching genetic background and generated by the same IMPC Centre. Mutant and wt mice were phenotyped in the same manner based on the IMPReSS protocol, and detailed experiment characteristics captured in the procedure metadata. For data analysis, mutants were matched with wt animals from the same center that used the same metadata. Detailed experimental protocols on the IMPC phenotyping procedures are available for general access at www.mousephenotyping.org/IMPReSS.

Animal experimentation

IMPC high-throughput phenotyping pipeline characterizes a mouse by a series of standardised and validated set of tests underpinned by standard operating procedures (SOPs). The IMPReSS database (https://www.mousephenotype.org/impress) defines the data that is to be collected as well as the experimental design, detailed procedural information, age of the mice, significant metadata parameters and data QC for IMPC pipeline tests. Animals are not randomly allocated

to experiment groups, rather we rely on Mendelian inheritance to provide the randomization method. Still, varieties of approaches are taken at different institutes to minimize bias such as order effects including alternate animal order, cage casual randomization and casual randomization within a cage. There were no consistent approaches to blinding for data collection and annotation across the institutes within IMPC. These issues are discussed with regard to the ARRIVE guidelines at http://www.mousephenotype.org/about-impc/arrive-guidelines.

Housing and husbandry

Housing and husbandry data are captured for each IMPC Centre as described in Karp et al.[7] and available on the IMPC portal (http://www.mousephenotype.org/about-impc/arrive-guidelines). Moreover, pertinent metadata parameters for each IMPC center are available from the portal including, cage-type, caging density, bedding and enrichment details, feed constituents, lighting regimes, temperature and humidity of animal holding rooms, and full strain nomenclature.

Generation of mutant mouse strains

The IMPC systematically phenotypes mice that are homozygous for a single-gene knockout or heterozygous when homozygotes are lethal or sub-viable. Mouse production was coordinated by iMits (https://www.mousephenotype.org/imits/).

The gene-targeting strategies that are used can be accessed via http://www.mousephenotype.org/about-ikmc/targeting-strategies. For every IMPC deleted gene, specifics on the targeting strategy can be obtained through a gene search on the IMPC website (http://www.mousephenotype.org) and use of the “Order Mouse and ES Cells” tab.

In the case of single copy genes, hemizygous knockout mice are studied. IMPC mouse models are available to the research community via the IMPC website.

As a high throughput project, the sample size is relatively low with a target number of knockout animals being processed of 14 (7 per sex). This number was arrived at after a community wide debate that involved statisticians, biologists and project managers to find the lowest number that would consume the least amount of resources while achieving the goal of detecting phenotype abnormalities in a strain. At times, practical issues might limit the number of animals it is possible to test such as viability issues or the difficulty in administering a test. As such, each time data are shown, the number of animals phenotyped per sex per genotype is listed with the graphical visualization of the data. In a high throughput environment, replication of individual lines is not cost effective (see https://www.mousephenotype.org/about-impc/animal-welfare/arrive-guidelines/ for more details).

Approved animal protocols

All animal work described in this study was carried under the auspice of approved animal protocols: Baylor College of Medicine (#AN-5896), German Mouse Clinic Helmholtz Zentrum München (#144-10, 15-168), Institut Clinique de la Souris Mouse Clinical Institute (#4789-2016040511578546v2), Medical Research Council Harwell[8/3384], Nanjing University (#NRCMM9), Rikagaku Kenkyūjo Tsukuba Institute (#Exp11-011, 12-011, 13-011, 14-009, 14-017, 15-009, 16-008), The Centre for Phenogenomics (#0153, 0275, 0277, 0279), The Jackson Laboratory (#11005) , and the University of California Davis (#20863).

Ethical approval

Ethical review s, licensing and accrediting bodies to breed mice and collect phenotyping data were followed by all IMPC Centers and reflected the national legislation under which the centers operate. Details of their ethical review bodies and licenses are provided upon request. The housing and husbandry capture form is an institute overview form to capture how the animals are housed and cared for as the environment influences the observed phenotype. The questions have

been based on the requirements of the Animal Research: Reporting In Vivo Experiments guidelines (ARRIVE)[9], Gold Standard Publication Checklist (GSPC) reporting guidelines[10] and the GA passport (RSPCA 2010).

Comprehensive housing and husbandry information fully accessible via:

https://www.mousephenotype.org/impress/ProcedureInfo?action=list&procID=1413&pipeID=7.

The Welfare observations procedure is used for the recording of welfare issues as and when they occur. Observations are submitted using controlled vocabulary with optional submission of images and free-text comments to document the issues.

Comprehensive welfare information is fully accessible via:

https://www.mousephenotype.org/impress/ProcedureInfo?action=list&procID=1216&pipeID=7.

Data quality control

Data generated in each IMPC center are captured centrally by the Data Coordination Centre (DCC), where a team of data wranglers perform quality control (QC). The QC process involves data wranglers checking all data both manually and with automated methods. QC issues are raised through a QC web interface, where IMPC centers can respond to confirm it is an issue or alternatively that the data are correct. Each QC issue is then tracked with the corresponding data points until it has been corrected by the data contributing center. Once the data has passed QC, it is released to the Core Data Archive (CDA) at the European Bioinformatics Institute, through a regular release schedule. The data set analyzed here consists largely of data from the IMPC data release 10.1 (DR10.1) with a smaller set of pre-QC data from the DCC. For the purpose of this article, the ECG and TTE data in this smaller set were QC'd and manually curated.

Mapping to MP ontology terms

MP terms for the genes of interest were extracted from the MGI database (http://www.informatics.jax.org/) using the file (MGI_PhenoGenoMP.rpt), which contains

information on genes and their annotated phenotypes. In order to avoid circular discoveries, as the MGI database includes the IMPC phenotype data, IMPC entries were removed using internal filters.

Mutant mouse lines found to have phenotype hits were assigned by searching the IMPC database (version 10, released April, 2019) for Mammalian Phenotype (MP) terms as defined in the IMPReSS protocol[11]. ECG and ECHO related MP terms are embedded in cardiovascular system phenotype (MP:0005385), abnormal cardiovascular system morphology (MP:0002127) and abnormal cardiovascular system physiology (MP:0001544) and can be queried by the following link: ECG: www.mousephenotype.org/impress/ProcedureInfo?action=list&procID=932, and ECHO: www.mousephenotype.org/impress/ProcedureInfo?action=list&procID=654.

Data availability

All data generated or analyzed during this study are included in this published article and its supplementary information files. IMPC data is open accessed for public.

Single gene search:

https://www.mousephenotype.org/data/search.

For batch query please go to:

https://www.mousephenotype.org/data/batchQuery

Particular data release (DR) download:

http://ftp.ebi.ac.uk/pub/databases/impc/all-data-releases/.

Support for particular data release (DR – here we used DR10.1) download:

https://www.mousephenotype.org/help/programmatic-data-access/.

Personal request:

http://www.mousephenotype.org/contact-us.

Statistical methods

The workflow can be described in two major stages: 1. Data collection and 2. Statistical analysis. In the **data collection stage**, a set of 14 mice (7 male and 7 female) per gene for ECG and TTE, and the parameters wherein are measured in globally distributed centres under a unified experiment framework described in the IMPReSS (https://www.mousephenotype.org/impress). The resulting data is then tested for the quality control (QC) measures such as missing values, out of range values, mislabeled values and/or dates, etc. The datasets that pass the QC step are integrated into the IMPC data infrastructure for performing the statistical analysis as well as disseminated from the web portal https://www.mousephenotype.org.

In the **statistical analysis stage**, the raw data, here from IMPC data release 10.1 (June 2019), are passed through three analysis steps. The initial step consists of preparing individualized datasets for each mutant line by selecting the most appropriate control set on the basis of the experiment design elements such as the background strain, zygosity, metadata and so on. The second step consists of data filtering and third step describes the statistical analysis followed by storing and disseminating the results. Detailed information on all these steps are outlined on: https://www.mousephenotype.org/help/data-analysis/from-parameter-to-phenotype.

The prepared datasets are then analyzed separately using an optimized, windowed[12] linear mixed model[13] with $Genotype$, Sex, $Genotype \times Sex$ interaction and $Bodyweight$ in the fixed effect term of the model and $Batch$, defined as the date on which the measurements were performed, in the random effect. The term "windowed" and "optimized" refer respectively to the selection of the most appropriate local controls in time for the mutants; and backward elimination approach to remove the variables that are not significant (at the level of $\mathrm{q-value} < 0.05$) in the saturated model below

$$Response\ (parameter) = Genotype + Sex + Genotype \times Sex\ (interaction\ term) + Bodyweight + Batch\ (random\ effect).$$

Pharos

Pharos (https://pharos.nih.gov), a multimodal web interface that presents the data from **Target Central Resource Database** (TCRD)[14], which collates many heterogeneous gene/protein datasets was used to query the mouse genes. Combining mouse and human phenotype data led to classification of "known" and "unknown" gene targets[15]. In this analysis, IMPC information was excluded to avoid any bias.

OMIM and Orphanet

Online Mendelian Inheritance in Man (OMIM, www.omim.org) and Orphanet (www.orpha.net) were queried for Pharos confirmation. Top-level term, HP_0001627: Abnormality of cardiac morphology; Abnormality of the heart; Abnormally shaped heart; Cardiac abnormality; Cardiac anomalies; Congenital heart defect; Congenital heart defects, was selected for HP / gene alignment with human heart disease information in these two databases. The intersection assessment process was completed on October 11, 2020.

Pediatric Cardiac Genomics Consortium Overview

The Pediatric Cardiac Genomics Consortium (PCGC) is a group of clinical research teams, supported by appropriate cores and research infrastructure, collaborating to identify genetic causes of human congenital heart disease (CHD) and to relate genetic variants present in the congenital heart disease patient population to clinical outcomes. The Bench to Bassinet Program is a major effort launched by the National Heart, Lung, and Blood Institute to learn more about how the heart develops and why children are born with heart problems (https://benchtobassinet.com). To align knockout strains to PCGC patient information, data was shared and further investigated.

The 100,000 Genomes Project

The project has sequenced 100,000 genomes from around 85,000 people. Participants are NHS patients with a rare disease, plus their families, and patients with cancer[16,17]. On 31st September 2018, recruitment to the rare disease pathway of the 100,000 Genomes Project closed. DNA samples from 61,282 rare disease patients and family members have been deposited in the UK Biobank and 87,231 whole genome sequences have been produced, from rare disease and cancer patients. To align knockout strains to the 100,000 genomes project data cases with congenital heart disease were selected *a priori*. Familial congenital heart disease encompasses well-defined inclusion criteria. Congenital heart disease AND one or more of the following: one or more first-degree relative with congenital heart disease OR parental consanguinity. Individuals with severe or syndromic disease or with consanguinity and a pedigree in keeping with autosomal recessive inheritance were recruited according to standard guidance, typically as trios. Disease status of apparently unaffected participants was determined according to standard clinical practice to detect cryptic disease. In other cases, unaffected individuals were not recruited. Recruitment in such families favored multiplex families over single isolated cases. Singleton recruits did not contribute to the overall singleton monitoring metrics applied to GMCs. Familial congenital heart disease exclusion criteria: Recognized syndromic presentation (e.g. Noonan syndrome). Likely causative environmental insult during gestation. This information was derived from the official definition based on the Rare Disease Conditions Eligibility Criteria.

Network analysis

Network analysis was performed using CIDeR (http://mips.helmholtz-muenchen.de/cider/), a publicly available, manually curated, integrative database of metabolic and neurological disorders. The resource provides structured information on more than 109,000 experimentally validated interactions between molecules, bioprocesses and environmental factors extracted from scientific literature. Systematic annotation and interactive graphical representation of disease

networks make CIDeR a versatile knowledge base for biologists, analyses of large-scale data and systems biology approaches[2].

Manual curation

Manual curation was conducted via manual curation of scientific articles and additional resources, which are referenced by PubMed identifier or web link in the table. The annotation process was completed in October 2020. The literature search was performed by searching OMIM (PMID: 30445645), PubMed (PMID: 30395293), PubMed Central (PMID: 30395293) and Google Scholar.

Gene-disease network enrichment analysis - Hairball analysis

We conducted the gene-disease 'hairball' network enrichment analysis for the 486 human orthologues of mouse 'unknown' cardiac genes using the NetworkAnalyst automatic tool (http://www.networkanalyst.ca)[18]. The gene-disease and gene-phenotype associations were obtained from the DisGenet database[19].

Gene expression analysis

The expression levels between positive (P, genes with significant phenotypes) and negative (N, without significant phenotypes) gene groups from our mouse study at different developmental stages, stratified by procedure (ECG or TTE). From a total of 3894 genes, n=3444 had expression data in cardiac tissue and n=450 did not. In addition, a comparison was made between known congenital heart disease (CHD)[20]. To assess the gene expression levels in both mouse and human heart, a publicly available transcriptomics atlas was used[21]. The RPKM (Reads Per Kilobase Million) matrices for mouse and human heart tissue were accessed through ArrayExpress database with accessions E-MTAB-6798 and E-MTAB-6814, respectively. Gene expression levels were averaged among samples in the different development stages of the heart

as follow; i) mouse: development (E10.5-E13.5), maturation, (E14.5-E18.5) and postnatal (P0-P63), ii) human: development (4wpc-8wpc), maturation (9wpc-20wpc) and postnatal (newborn-adulthood). Human gene IDs were mapped 1:1 (orthologous) to Ensembl mouse gene IDs. Comparison between known congenital heart disease (CHD) genes[20], ECG/TTE positive and ECG/TTE negative genes was performed using a Wilcoxon rank-sum test, stratified further by developmental stages.

Permutation procedure

A permutation-based test was performed to evaluate the impact of the size difference between positive and negative gene groups (e.g., larger number of negative genes compared to positive ones). The negative group was randomly down sampled to generate 50,000 sub-groups of equal size compared to the positive group. To compute an empirical p-value, the number of times the averaged expression was observed higher in negative group than positive group (fails) was divided by the number of permutations (50,000). The permutation-based analysis was stratified by procedure (ECG and TTE) and developmental stages (development, maturation and postnatal).

Literature:

1 Dickinson, M. E. *et al.* High-throughput discovery of novel developmental phenotypes. *Nature* **537**, 508-514, doi:10.1038/nature19356 (2016).

2 Lechner, M. *et al.* CIDeR: multifactorial interaction networks in human diseases. *Genome biology* **13**, R62, doi:10.1186/gb-2012-13-7-r62 (2012).

3 Skarnes, W. C. *et al.* A conditional knockout resource for the genome-wide study of mouse gene function. *Nature* **474**, 337-342, doi:10.1038/nature10163 (2011).

4 Lloyd, K. d. J., P. . The knockout mouse project (KOMP) repository. *Transgenic Research* **19**, 338–339 (2010).

5 Gertsenstein, M. *et al.* Efficient generation of germ line transmitting chimeras from C57BL/6N ES cells by aggregation with outbred host embryos. *PloS one* **5**, e11260, doi:10.1371/journal.pone.0011260 (2010).

6 Birling, M.-C. *et al.* (bioRxiv, 2019).

7 Hrabe de Angelis, M. *et al.* Analysis of mammalian gene function through broad-based phenotypic screens across a consortium of mouse clinics. *Nature genetics* **47**, 969-978, doi:10.1038/ng.3360 (2015).

8 Kemp, J. P. *et al.* Phenotypic dissection of bone mineral density reveals skeletal site specificity and facilitates the identification of novel loci in the genetic regulation of bone mass attainment. *PLoS genetics* **10**, e1004423, doi:10.1371/journal.pgen.1004423 (2014).

9 Kilkenny, C., Browne, W. J., Cuthill, I. C., Emerson, M. & Altman, D. G. Improving bioscience research reporting: the ARRIVE guidelines for reporting animal research. *PLoS biology* **8**, e1000412, doi:10.1371/journal.pbio.1000412 (2010).

10 Hooijmans, C. R., Leenaars, M. & Ritskes-Hoitinga, M. A gold standard publication checklist to improve the quality of animal studies, to fully integrate the Three Rs, and to make systematic reviews more feasible. *Altern Lab Anim* **38**, 167-182, doi:10.1177/026119291003800208 (2010).

11 Bult, C. J., Blake, J. A., Smith, C. L., Kadin, J. A. & Richardson, J. E. Mouse Genome Database (MGD) 2019. *Nucleic acids research* **47**, D801-d806, doi:10.1093/nar/gky1056 (2019).

12 Haselimashhadi, H. *et al.* Soft windowing application to improve analysis of high-throughput phenotyping data. *Bioinformatics (Oxford, England)* **36**, 1492-1500, doi:10.1093/bioinformatics/btz744 (2020).

13 G.E.Gilbert. Linear Mixed Models: A Practical Guide Using Statistical Software. *J. Am. Stat. Assoc.* **103**, 427-428 (2009).

14 Nguyen, D. T. *et al.* Pharos: Collating protein information to shed light on the druggable genome. *Nucleic acids research* **45**, D995-d1002, doi:10.1093/nar/gkw1072 (2017).

15 Oprea, T. I. *et al.* Unexplored therapeutic opportunities in the human genome. *Nature reviews. Drug discovery* **17**, 317-332, doi:10.1038/nrd.2018.14 (2018).

16 Moss, C. & Wernham, A. The 100 000 Genomes Project: feeding back to patients. *BMJ (Clinical research ed.)* **361**, k2441, doi:10.1136/bmj.k2441 (2018).

17 Turnbull, C. *et al.* The 100 000 Genomes Project: bringing whole genome sequencing to the NHS. *BMJ (Clinical research ed.)* **361**, k1687, doi:10.1136/bmj.k1687 (2018).

18 Zhou, G. *et al.* NetworkAnalyst 3.0: a visual analytics platform for comprehensive gene expression profiling and meta-analysis. *Nucleic acids research* **47**, W234-w241, doi:10.1093/nar/gkz240 (2019).

19 Piñero, J. *et al.* The DisGeNET knowledge platform for disease genomics: 2019 update. *Nucleic acids research* **48**, D845-d855, doi:10.1093/nar/gkz1021 (2020).

20 Audain, E. *et al.* Integrative analysis of genomic variants reveals new associations of candidate haploinsufficient genes with congenital heart disease. *PLoS genetics* **17**, e1009679, doi:10.1371/journal.pgen.1009679 (2021).
21 Cardoso-Moreira, M. *et al.* Gene expression across mammalian organ development. *Nature* **571**, 505-509, doi:10.1038/s41586-019-1338-5 (2019).

Supplementary Figure 1:

Homozygous *Smo* knockouts died at/or around E9.5 from multiple abnormalities that included craniofacial defects and malformation of ventricles and atria. Panel a and c represent the C57BL/6N control E9.5 embryo, whereas panel b and d represent the *Smo* knockout embryo. Craniofacial defects are seen in panel b, whereas malformation of ventricles and atria is seen in panel d.

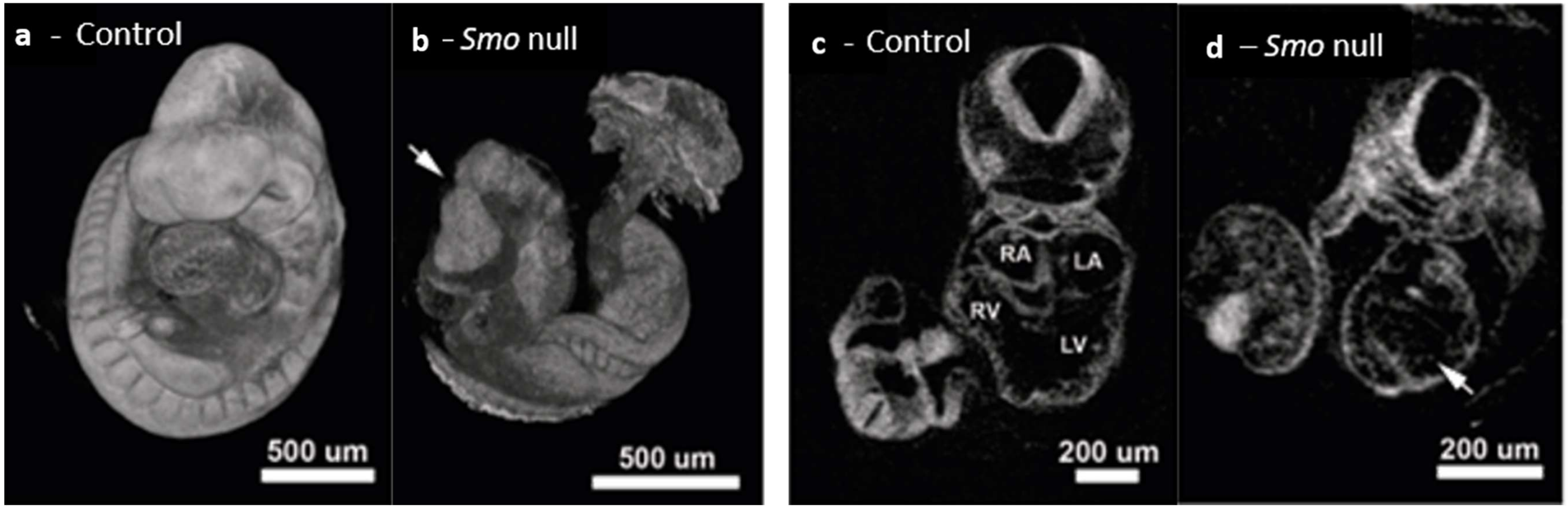

Supplement: Supplementary file 1 — Supplementary Methods and Fig. 1 [file 44161_2022_18_MOESM1_ESM.pdf]
