# Supplementary material for: Extensive identification of genes involved in congenital and structural heart disorders and cardiomyopathy
Source: Nat Cardiovasc Res. 2022 Feb 17;1(2):157–73. doi: 10.1038/s44161-022-00018-8 (PMC11358025; doi:10.1038/s44161-022-00018-8)
Supplement: Supplementary file 2 — Reporting Summary [file 44161_2022_18_MOESM2_ESM.pdf]

nature research

Corresponding author(s): Martin Hrabe de Angelis, PhD, Institute of Experimental Genetics, Helmholtz Center Munich, Ingolstaedter Landstr. 1, 85764 Neuherberg, Germany, hrabe@helmholtz-muenchen.de, +49 89 3187-3302

Last updated by author(s): 12-20-2021

Reporting Summary

Nature Research wishes to improve the reproducibility of the work that we publish. This form provides structure for consistency and transparency in reporting. For further information on Nature Research policies, see our Editorial Policies and the Editorial Policy Checklist.

Statistics

For all statistical analyses, confirm that the following items are present in the figure legend, table legend, main text, or Methods section.

n/a	Confirmed	
☐	☒	The exact sample size (n) for each experimental group/condition, given as a discrete number and unit of measurement
☐	☒	A statement on whether measurements were taken from distinct samples or whether the same sample was measured repeatedly
☐	☒	The statistical test(s) used AND whether they are one- or two-sided<br>*Only common tests should be described solely by name; describe more complex techniques in the Methods section.*
☐	☒	A description of all covariates tested
☐	☒	A description of any assumptions or corrections, such as tests of normality and adjustment for multiple comparisons
☐	☒	A full description of the statistical parameters including central tendency (e.g. means) or other basic estimates (e.g. regression coefficient) AND variation (e.g. standard deviation) or associated estimates of uncertainty (e.g. confidence intervals)
☐	☒	For null hypothesis testing, the test statistic (e.g. F, t, r) with confidence intervals, effect sizes, degrees of freedom and P value noted<br>*Give P values as exact values whenever suitable.*
☒	☐	For Bayesian analysis, information on the choice of priors and Markov chain Monte Carlo settings
☒	☐	For hierarchical and complex designs, identification of the appropriate level for tests and full reporting of outcomes
☒	☐	Estimates of effect sizes (e.g. Cohen's d, Pearson's r), indicating how they were calculated

Our web collection on statistics for biologists contains articles on many of the points above.

Software and code

Policy information about availability of computer code

Data collection

In the data collection stage, a set of 14 mice (7 male and 7 female) per gene for ECG and TTE, and the parameters wherein are measured in globally distributed centres under a unified experiment framework described in the IMPReSS (https://www.mousephenotype.org/impress). The resulting data is then tested for the quality control (QC) measures such as missing values, out of range values, mislabeled values and/or dates, etc. The datasets that pass the QC step are integrated into the IMPC data infrastructure for performing the statistical analysis as well as disseminated from the web portal https://www.mousephenotype.org.

Data analysis

In the statistical analysis stage, the raw data, here from IMPC data release 10.1 (June 2019), are passed through three analysis steps. The initial step consists of preparing individualized datasets for each mutant line by selecting the most appropriate control set on the basis of the experiment design elements such as the background strain, zygosity, metadata and so on. The second step consists of data filtering and third step describes the statistical analysis followed by storing and disseminating the results. Detailed information on all these steps are outlined on: https://www.mousephenotype.org/help/data-analysis/from-parameter-to-phenotype.
The prepared datasets are then analyzed separately using an optimized, windowed (Haselimashhadi, H. et al. Bioinformatics (Oxford, England) 36, 1492-1500, 2020) linear mixed model (G.E.Gilbert. J. Am. Stat. Assoc. 103, 427-428, 2009) with Genotype, Sex, Genotype×Sex interaction and Bodyweight in the fixed effect term of the model and Batch, defined as the date on which the measurements were performed, in the random effect. The term “windowed” and “optimized” refer respectively to the selection of the most appropriate local controls in time for the mutants; and backward elimination approach to remove the variables that are not significant (at the level of q-value<0.05) in the saturated model below
Response (parameter)= Genotype + Sex + Genotype×Sex (interaction term)+ Bodyweight + Batch (random effect).

For manuscripts utilizing custom algorithms or software that are central to the research but not yet described in published literature, software must be made available to editors and reviewers. We strongly encourage code deposition in a community repository (e.g. GitHub). See the Nature Research guidelines for submitting code & software for further information.

Data

Policy information about availability of data

All manuscripts must include a data availability statement. This statement should provide the following information, where applicable:

- Accession codes, unique identifiers, or web links for publicly available datasets
- A list of figures that have associated raw data
- A description of any restrictions on data availability

All data generated or analyzed during this study are included in this published article and its supplementary information files. IMPC data is open accessed for public.
Single gene search:
https://www.mousephenotype.org/data/search.
Batch query:
https://www.mousephenotype.org/data/batchQuery
Particular data release (DR) download:
http://ftp.ebi.ac.uk/pub/databases/impc/all-data-releases/.
Support for particular data release (DR – here we used DR10.1) download:
https://www.mousephenotype.org/help/programmatic-data-access/.

Field-specific reporting

Please select the one below that is the best fit for your research. If you are not sure, read the appropriate sections before making your selection.

☒ Life sciences ☐ Behavioural & social sciences ☐ Ecological, evolutionary & environmental sciences

For a reference copy of the document with all sections, see nature.com/documents/nr-reporting-summary-flat.pdf

Life sciences study design

All studies must disclose on these points even when the disclosure is negative.

Sample size	As a high throughput project, the sample size is relatively low with a target number of knockout animals being processed of 14 (7 per sex) for the standard Early Adult phenotyping. This number was arrived at after a community wide debate that involved statisticians, biologists and project managers to find the lowest number that would consume the least amount of resources while achieving the goal of detecting phenotype abnormalities in a strain. At times, practical issues might limit the number of animals it is possible to test, such as viability issues or the difficulty in administering a test. As such, each time data are shown, the number of animals phenotyped per sex per genotype is listed with the graphical visualisation of the data. See https://www.mousephenotype.org/about-impc/animal-welfare/arrive-guidelines/) for more details. See https://www.mousephenotype.org/about-impc/animal-welfare/arrive-guidelines/) for more details.
Data exclusions	IMPC data is tested with quality control measures such as missing values, out of range values, mislabeled values and/or dates (https://www.mousephenotype.org/impress). In general, no data point will be excluded from the analysis, unless there is clear evidence of e.g. technical failure of the experimental machine. For many of the parameters we have pre-established threshold values as indicators for invalid measurements. If the data point reaches the criteria we have to expect that there was an undetectable failure, case where the data point is replaced by a status code.
Replication	In a high throughput environment, replication of individual lines is not cost effective. Instead, multiple IMPC centres generated and characterised the same six reference knockout lines that present a wide range of phenotypes based on previously published research (Analysis of mammalian gene function through broad-based phenotypic screens across a consortium of mouse clinics; Nat Genet. 2015 Sep;47(9):969-978.; PMID: 26214591). See https://www.mousephenotype.org/about-impc/animal-welfare/arrive-guidelines/) for more details.
Randomization	Within the analysis we consider the mouse as the experimental unit. Groups of mice are assigned according to the sex and genotype of the animals. The random allocation of mice to experimental group (wildtype versus mutant, male and female) is driven by Mendelian Inheritance. Unlike most experiments, we cannot randomly allocate animals to experiment groups; Still, varieties of approaches are taken at different institutes to minimize bias such as order effects including alternate animal order, cage casual randomization and casual randomization within a cage. See https://www.mousephenotype.org/about-impc/animal-welfare/arrive-guidelines/) for more details.
Blinding	There were no consistent approaches to blinding for data collection and annotation across the institutes within IMPC. In most tests, blinding is not needed, as the experimenter has no influence on the results of these tests, since the results are directly recorded by the machine. In the tests, where the experimenter might have an influence on the measurements, the experimenter is blinded, and we have SOP's prescribing how this will be ensured for each single test separately. In addition, we record metadata for each data point and monitor the influence of these metadata over time. See https://www.mousephenotype.org/about-impc/animal-welfare/arrive-guidelines/)

Reporting for specific materials, systems and methods

We require information from authors about some types of materials, experimental systems and methods used in many studies. Here, indicate whether each material, system or method listed is relevant to your study. If you are not sure if a list item applies to your research, read the appropriate section before selecting a response.

Materials & experimental systems

n/a	Involved in the study
☒	☐ Antibodies
☒	☐ Eukaryotic cell lines
☒	☐ Palaeontology and archaeology
☐	☒ Animals and other organisms
☒	☐ Human research participants
☐	☒ Clinical data
☒	☐ Dual use research of concern

Methods

n/a	Involved in the study
☐	☒ ChIP-seq
☒	☐ Flow cytometry
☒	☐ MRI-based neuroimaging

Antibodies

Antibodies used: n/a

Validation: n/a

Eukaryotic cell lines

Policy information about cell lines

Cell line source(s): n/a

Authentication: n/a

Mycoplasma contamination: n/a

Commonly misidentified lines (See ICLAC register): n/a

Palaeontology and Archaeology

Specimen provenance: n/a

Specimen deposition: n/a

Dating methods: n/a

☐ Tick this box to confirm that the raw and calibrated dates are available in the paper or in Supplementary Information.

Ethics oversight: n/a

Note that full information on the approval of the study protocol must also be provided in the manuscript.

Animals and other organisms

Policy information about studies involving animals; ARRIVE guidelines recommended for reporting animal research

Laboratory animals: All mutants were young adult mice (12 weeks of age) on the C57BL/6N background. In general both sexes were analysed. Mice were maintained with water and standard mouse chow according to the housing conditions of the phenotyping center and the applicable law of the respective country. The SOPs of the phenotyping test and housing conditions of the each center involved in the study you find at https://www.mousephenotype.org/impress/pipelines.

Wild animals: n/a

Field-collected samples: n/a

Ethics oversight: IMPC follows the ARRIVE guidelines. All IMPC institutes that breed mice and collect phenotyping data are guided by their individual ethical review panels and licensing and accrediting bodies, reflecting the national legislation under which they operate. Cardiovascular mouse phenotyping was carried out under the auspice of the following animal protocols: Baylor College of Medicine (#AN-5896), German Mouse Clinic Helmholtz Zentrum München (#144-10, 15-168), Institut Clinique de la Souris Mouse Clinical Institute (#4789-2016040511578546v2), Medical Research Council Harwell 95/3384, Nanjing University (#NRCMM9), Rikagaku Kenkyūjo Tsukuba Institute (#Exp11-011, 12-011, 13-011, 14-009, 14-017, 15-009, 16-008), The Centre for Phenogenomics (#0153, 0275, 0277, 0279), The Jackson Laboratory (#11005) , and the University of California Davis (#20863).

Note that full information on the approval of the study protocol must also be provided in the manuscript.

Human research participants

Policy information about studies involving human research participants

Population characteristics	n/a
Recruitment	n/a
Ethics oversight	n/a

Note that full information on the approval of the study protocol must also be provided in the manuscript.

Clinical data

Policy information about clinical studies

All manuscripts should comply with the ICMJE guidelines for publication of clinical research and a completed CONSORT checklist must be included with all submissions.

Clinical trial registration	n/a
Study protocol	PCGC data were generated by the Pediatric Cardiac Genetics Consortium (PCGC), under the auspices of the National Heart, Lung, and Blood Institute's Bench to Bassinet Program <http://www.benchtobassinet.org/>. The 100,000 Genomes Project uses data provided by patients and collected by the National Health Service as part of their care and support. The National Genomics Research and Healthcare Knowledgebase v5, Genomics England. doi:10.6084/m9.figshare.4530893.v5. 2019. UK Biobank data set from the Neale Lab - http://www.nealelab.is/data,
Data collection	Clinical data used were previously published and no clinical data collection was performed by the authors of this manuscript.
Outcomes	n/a

Dual use research of concern

Policy information about dual use research of concern

Hazards

Could the accidental, deliberate or reckless misuse of agents or technologies generated in the work, or the application of information presented in the manuscript, pose a threat to:

No	Yes	
☒	☐	Public health
☒	☐	National security
☒	☐	Crops and/or livestock
☒	☐	Ecosystems
☒	☐	Any other significant area

Experiments of concern

Does the work involve any of these experiments of concern:

No	Yes	
☒	☐	Demonstrate how to render a vaccine ineffective
☒	☐	Confer resistance to therapeutically useful antibiotics or antiviral agents
☒	☐	Enhance the virulence of a pathogen or render a nonpathogen virulent
☒	☐	Increase transmissibility of a pathogen
☒	☐	Alter the host range of a pathogen
☒	☐	Enable evasion of diagnostic/detection modalities
☒	☐	Enable the weaponization of a biological agent or toxin
☒	☐	Any other potentially harmful combination of experiments and agents

ChIP-seq

Data deposition

☒ Confirm that both raw and final processed data have been deposited in a public database such as GEO.

☐ Confirm that you have deposited or provided access to graph files (e.g. BED files) for the called peaks.

Data access links<br>*May remain private before publication.*	We did not perform our own ChipSeq Data. We accessed the ChEA2016 dataset of publicly available ChIP-Seq experiments PMID: 27141961
Files in database submission	Supplementary_Table_2
Genome browser session<br>(e.g. UCSC)	n/a

Methodology

Replicates	n/a
Sequencing depth	n/a
Antibodies	n/a
Peak calling parameters	n/a
Data quality	n/a
Software	n/a

Flow Cytometry

Plots

Confirm that:

☐ The axis labels state the marker and fluorochrome used (e.g. CD4-FITC).

☐ The axis scales are clearly visible. Include numbers along axes only for bottom left plot of group (a 'group' is an analysis of identical markers).

☐ All plots are contour plots with outliers or pseudocolor plots.

☐ A numerical value for number of cells or percentage (with statistics) is provided.

Methodology

Sample preparation	n/a
Instrument	n/a
Software	n/a
Cell population abundance	n/a
Gating strategy	n/a

☐ Tick this box to confirm that a figure exemplifying the gating strategy is provided in the Supplementary Information.

Magnetic resonance imaging

Experimental design

Design type	n/a
Design specifications	n/a
Behavioral performance measures	n/a

Acquisition

Imaging type(s)	n/a
Field strength	n/a
Sequence & imaging parameters	n/a
Area of acquisition	n/a

Diffusion MRI ☐ Used ☐ Not used

Preprocessing

Preprocessing software	n/a
Normalization	n/a
Normalization template	n/a
Noise and artifact removal	n/a
Volume censoring	n/a

Statistical modeling & inference

Model type and settings	n/a
Effect(s) tested	n/a

Specify type of analysis: ☐ Whole brain ☐ ROI-based ☐ Both

Statistic type for inference (See Eklund et al. 2016)	n/a
Correction	n/a

Models & analysis

n/a	Involved in the study
☒	☐ Functional and/or effective connectivity
☒	☐ Graph analysis
☒	☐ Multivariate modeling or predictive analysis
